# Trans-omics analyses identify the biochemical network of LPCAT1 associated with coronary artery disease

**DOI:** 10.1186/s40364-025-00821-y

**Published:** 2025-08-20

**Authors:** Paul Wei-Che Hsu, Chi-Hsiao Yeh, Chi-Jen Lo, Tsung-Hsien Tsai, Yun-Hsuan Chan, Yi-Ju Chou, Ning-I Yang, Mei-Ling Cheng, Wayne Huey-Herng Sheu, Chi-Chun Lai, Huey-Kang Sytwu, Ting-Fen Tsai

**Affiliations:** 1 Institute of Molecular and Genomic Medicine, National Health Research Institutes, Miaoli, Taiwan; 2 Department of Thoracic and Cardiovascular Surgery, Chang Gung Memorial Hospital, Linkou, Taoyuan, Taiwan; 3 Community Medicine Research Center, Chang Gung Memorial Hospital, Keelung, Taiwan; 4 College of Medicine, Chang Gung University, Taoyuan, Taiwan; 5 Metabolomics Core Laboratory, Healthy Aging Research Center, Chang Gung University, Taoyuan, Taiwan; 6 Advanced Tech BU, Acer Inc, New Taipei City, Taiwan; 7 Division of Cardiology, Department of Internal Medicine, Chang Gung Memorial Hospital, Keelung, Taiwan; 8 Clinical Metabolomics Core Laboratory, Chang Gung Memorial Hospital, Taoyuan, Taiwan; 9 Department of Biomedical Sciences, College of Medicine, Chang Gung University, Taoyuan, Taiwan; 10 Division of Endocrinology and Metabolism, Department of Internal Medicine, Taipei Veterans General Hospital, Taipei, Taiwan; 11 School of Medicine, National Yang Ming Chiao Tung University, Taipei, Taiwan; 12 Department of Ophthalmology, Chang Gung Memorial Hospital, Keelung, Taiwan; 13 National Institute of Infectious Diseases and Vaccinology, National Health Research Institutes, Miaoli, Taiwan; 14 Department & Graduate Institute of Microbiology and Immunology, National Defense Medical Center, Taipei, Taiwan; 15 Department of Life Sciences and Institute of Genome Sciences, National Yang Ming Chiao Tung University, Taipei, Taiwan; 16 Aging and Health Research Center, National Yang Ming Chiao Tung University, Taipei, Taiwan

**Keywords:** Coronary artery disease (CAD), LPCAT1, Single-nucleotide polymorphism (SNP), Metabolomics, Machine learning, Biomarker

## Abstract

**Background:**

Coronary artery disease (CAD) remains a leading cause of mortality in developed nations. While previous genome-wide association studies have identified single-nucleotide polymorphisms (SNPs) linked to CAD, their impact on disease progression requires trans-omics validation.

**Methods:**

This study merges whole genome SNP analysis and metabolomic profiling to distinguish CAD patients from high-risk and healthy individuals. A cross-sectional study was conducted, enrolling participants from the Northeastern Taiwan Community Medicine Research Cohort, which spans the period between August 2013 and November 2020. A total of 781 participants were included in the study and categorized into three groups: control (*n* = 271), high-risk (*n* = 363), and CAD (*n* = 147) groups, following a stratification protocol. The study integrated K-clustering of metabolomics and SNP datasets. Subsequently, a machine-learning (ML)-assisted prediction model was developed specifically for CAD identification.

**Results:**

Four significant findings emerged. Firstly, plasma levels of phospholipids decline from healthy controls to high-risk individuals and then decline further among CAD patients. This indicates that plasma phospholipids have potential as biomarkers and implies that they have a role in CAD progression. Secondly, five genes are linked to lipidomic alterations via their top-ranking among CAD-associated SNPs. Thirdly, a specific LPCAT1 haplotype is associated with CAD using a trans-omics approach. Lastly, an ML-assisted trans-omics prediction model for CAD was developed, which achieves an area under the curve of 0.917, with LPCAT1 among the 16 top-ranked predictive features.

**Conclusion:**

This study highlights the usefulness of a multi-omics signature when discriminating CAD patients and suggests that abnormalities in phospholipid metabolism are influenced by LPCAT1 genetic variants. Our findings underscore the potential of multi-omics approaches to our understanding and identification of critical factors in CAD development.

**Trial registration number and date of registration:**

ClinicalTrials.gov Identifier: NCT04839796; Aug 2013.

**Supplementary Information:**

The online version contains supplementary material available at 10.1186/s40364-025-00821-y.

## Introduction

Coronary artery disease (CAD) remains the primary global cause of mortality and thus there is a need for a deeper understanding of its origins. This should lead to the development of more effective treatments. Genome-wide association studies (GWAS) focusing on the CAD phenotypes over the past decade have identified over 300 chromosomal loci significantly associated with the disease [1–5]. Remarkably, more than 90% of these common risk variants are situated outside of protein-coding regions [6], and these variants are only associated with about 25% of the disease’s heritability [7]. Additionally, trans-omics analysis, which amalgamates metabolic profiles with genomics, transcriptomics, proteomics, and clinical data, can offer more comprehensive datasets. Leveraging these approaches allows for the exploration of the underlying biological networks that influence susceptibility to a given disease [8]. By combining genetic association studies with the impact of various metabolites on biological pathways, the trans-omics analysis approach provides a more holistic perspective [9]. Notably, the integration of multiple layers of data in multi-omics studies has been shown to result in more dependable and more robust outcomes [10].

Metabolites encapsulate the outcomes of pathological processes and reflect the influences of genomic, epigenomic, and environmental factors [11, 12]. Within biomedical research, numerous plasma metabolites have emerged as pivotal predictors and risk factors for complex diseases, notably Alzheimer’s disease [13], diabetes [14], and CAD [7, 15, 16]. In the realm of CAD research specifically, this identification of metabolites across various pathways has unveiled a number of potential novel clinical biomarkers [17, 18].

Two distinct approaches have been employed to merge multiple omics datasets in order to unveil CAD risk factors. The first method, a multi-dimensional approach, such as network-based integration, uses high-dimensional data to impartially discern pathways causally linked to CAD from a comprehensive multi-omics viewpoint. Alternatively, the second approach targets specific genetic pathways associated with downstream omics data that are directly implicated in CAD development [7, 18, 19]. In this context, our hypothesis proposes that integrating metabolomics with genomics should be able to identify novel mechanisms or potential risk factors associated with CAD pathogenesis. To explore this, we conducted a genome-wide SNP analysis coupled with a targeted metabolomic assessment using three cohorts, namely a CAD cohort comprising individuals from cardiac catheterization (the CAD group), a CAD high-risk group, and a healthy control group. Our findings revealed that genetic loci associated with lipidome levels have an augmented CAD risk. Thus, by employing this multi-platform omics approach, we were able to identify a lipid metabolism pathway that is intricately linked with CAD progression.

## Methods

The authors declare that all supporting data, methods, and materials are available within the main body of the manuscript and in the online supplementary data.

### Study subjects

**Fig. 1:**
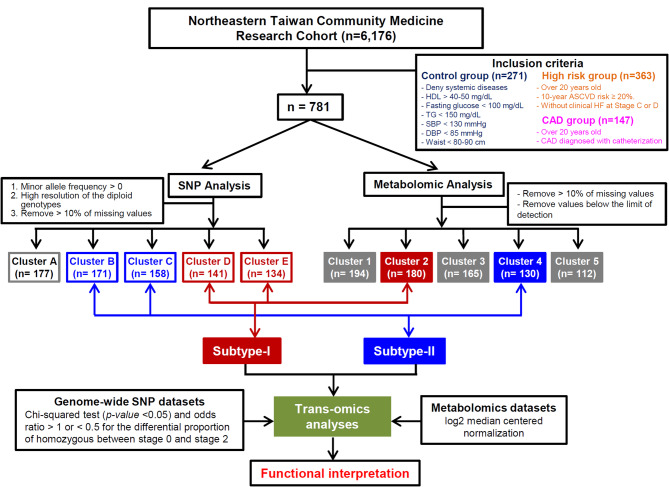
Flowchart of the study protocol. Three groups of subjects were included in this study. Cluster analyses of the genomic and metabolomic data separated the study subjects into five genetic and five metabolomic clusters. Trans-omics comparison identified two distinct patterns of subjects. CAD, coronary artery disease; HDL, high-density lipoprotein; TG, triglyceride; SBP, systolic blood pressure; DBP, diastolic blood pressure; ASCVD, atherosclerotic cardiovascular disease; HF, heart failure; SNP, single nucleotide polymorphism

A total of 781 subjects from the initially recruited 6,176 participants in the Northeastern Taiwan Community Medicine Research Cohort (ClinicalTrials.gov Identifier: NCT04839796, from July 2019 to November 2020; Fig. 1) were included in this study. All participants underwent a comprehensive clinical examination, blood tests, and a detailed personal and medical history assessment at the Department of Cardiology, Chang Gung Memorial Hospital, Keelung. By incorporating both a high-risk group and a CAD group, the potential confounding factors inherent to the study were mitigated. The disparities observed between the high-risk and CAD groups compared to the control group were thus able to attenuate the effects of any underlying coexisting conditions, allowing the primary focus to be on the CAD. The CAD group comprised individuals demonstrating angiographic evidence showing significant (> 75%) coronary artery stenosis post-administration of intracoronary nitroglycerine (50–200 µg), individuals with a history of percutaneous coronary revascularization or individuals who had undergone coronary artery bypass grafting. The high-risk group consisted of individuals whose calculated 10-year and lifetime risk of atherosclerotic cardiovascular disease (ASCVD) equaled or exceeded 20% based on established criteria [20, 21], but without any history of angina pectoris or myocardial infarction. All participants provided informed and signed consent for their participation in this study. The criteria for inclusion in the control group encompassed a number of factors, namely the absence of major systemic diseases, various specific fasting measurements including high-density lipoprotein (HDL) levels (> 40 mg/dL for males, > 50 mg/dL for females), fasting glucose levels (< 100 mg/dL), fasting triglyceride (TG) levels (< 150 mg/dL), systolic blood pressure (SBP) below 130 mmHg, diastolic blood pressure (DBP) below 85 mmHg, and a waist circumference below 80 cm for females or 90 cm for males. The study protocol adheres to the ethical guidelines outlined in the 1975 Declaration of Helsinki and received approval from the Institutional Review Board of Chang Gung Medical Foundation. (IRB No: 201800686B0, 202000077B0A3, and 202101810B0D001). Informed consent was obtained from all participants in the study.

### Clinical assessment

Upon recruitment, all participants underwent an extensive assessment involving a comprehensive personal history review, a thorough physical examination, the completion of a detailed questionnaire, and a range of biochemical tests, as previously described [21]. Blood pressure was assessed by taking the average of two seated measurements, while heart rate was determined via a resting 12-lead electrocardiogram. Body mass index (BMI) was calculated as weight divided by height squared (kg/m²). Regarding the blood pressure measurements, hypertension was deemed present if blood pressure exceeded 140/90 mmHg or if the participant was receiving antihypertensive medications for this condition. The aim of this comprehensive evaluation was to ensure a meticulous examination of the cardiovascular health and associated risk factors within the study population [22]. Diabetes mellitus was characterized by a fasting glucose level of ≥ 126 mg/dL, a glycohemoglobin level of ≥ 6.5%, a random glucose level of ≥ 200 mg/dL, or the use of hypoglycemic medications. Chronic kidney disease (CKD) was defined according to the National Kidney Foundation: Kidney Disease Outcomes Quality Initiative classification for CKD. This determination involved the presence of persistent proteinuria or a reduced estimated glomerular filtration rate (< 60 mL/min/1.73 m²), which was calculated using the abbreviated Modification of Diet in Renal Disease equation [23]. Proteinuria was identified if the urine protein to creatinine ratio equaled or exceeded 150 mg/g. A history of CAD was determined based on various indicators, namely the presence of angina pectoris during a positive exercise test, a history of myocardial infarction, angiographic evidence indicating significant (> 75%) coronary artery stenosis after intracoronary nitroglycerine administration (50–200 µg), or a history of undergoing percutaneous coronary revascularization or coronary artery bypass grafting. The current smoking status was classified as individuals who had smoked more than 100 cigarettes during their lifetime and had smoked within one month before their enrollment in the study.

### Targeted metabolite analysis using the p180 kit

Blood samples were collected using EDTA-treated tubes when the participants were recruited. Following centrifugation for a minimum of 15 minutes, the plasma component was isolated, frozen, and then shipped on dry ice to our hospital’s core laboratory center. Subsequently, these samples were stored at -80 °C to maintain stability before undergoing analysis using the AbsoluteIDQ p180 kit (BIOCRATES Life Sciences AG, Austria). For analysis, a 10 µL plasma sample was prepared according to the manufacturer’s instructions. The metabolites of amino acids and biogenic amines were quantified using liquid chromatography coupled with tandem mass spectrometry, while the lipid profile was assessed using flow injection analysis coupled with tandem mass spectrometry. The latter analysis was conducted in the positive electrospray ionization mode and employed multiple reaction monitoring on a TQS mass spectrometer (Waters Corp., Milford, USA). The MetIQ software package (Biocrates Life Science AG, Innsbruck, Austria) was used to facilitate the automatic calculation of metabolite concentrations, and the results were expressed in micromolar (µM) units [24]. The MetIQ software confirmed that the measured values of internal standards (D_8_-Val, D_7_-Pro, D_7_-ADMA, D_6_-Orn, D_5_-Phe, D_5_-Gln, D_4_-Tyr, D_4_-Serotonin, D_4_-Putresine, D_4_-Ala, D_3_-Ser, D_3_-Sarcosine, D_3_-Met, D_3_-Glu, D_3_-Asp, Creatinine, ^15^N_2_-Trp, ^15^N_2_-Asn, ^15^N_2_-Arg, ^13^C_6_-Ile, ^13^C_6_-His, ^13^C_4_-^15^N-Thr, ^13^C_2_-Taurine, ^13^C_2_-^15^N-Gly, and ^13^C-D_4_-Cit) and quality control samples (low QC, medium QC, and high QC) adhere to the predefined tolerance ranges. Altogether 143 targeted metabolites (21 amino acids, 9 biogenic amines, 15 acylcarnitines, 84 phospholipids, and 14 sphingolipids; Table S1) were measured using the unit variance method, which scales each variable by dividing by its standard deviation [25]. The data were processed by orthogonal partial least squares discriminant analysis (OPLS-DA) using the SIMCA-P 13.0 software (Umetrics, Umeå, Sweden). The 95% critical limit of the Hotelling *T*^2^ was defined by the ellipse in the score plot. The OPLS-DA model was constructed based on the internal 7-fold cross-validation.

### Whole-genome SNP analysis

The genotypes of the subjects were analyzed using the Axiom™ Genome-Wide TWB 2.0 array using genomic DNA isolated from white blood cells. Following this analysis, the SNPs were filtered sequentially based on a minor allele frequency < 0.01, deviation from Hardy-Weinberg equilibrium in the controls (*p* < 1 × 10⁻⁶), and a missing rate > 10%. For the remaining missing SNPs, imputation was performed by assigning the median allele observed across the dataset. This filtering process resulted in the removal of SNPs from an initial pool of 686,478 to leave a total of 370,394 SNPs that were eligible for subsequent analysis (Figure S1). Sample-level quality control confirmed that all individuals met Thermo Fisher Scientific’s genotyping standards (average call rate ≥ 98.5%), showed no heterozygosity outliers, and were unrelated (PI_HAT mean = 0.175, PLINK).

### Data normalization and K-means clustering of the various metabolites and SNPs

To ensure comparability across metabolites, missing values were imputed using the median, and zeros were replaced with 0.001 to facilitate log transformation. Each metabolite’s concentration across the 781 subjects was then normalized by dividing by its median and converted to a log scale. To standardize the varying concentrations of different metabolites, each metabolite’s concentration across the 781 subjects was normalized by dividing it with the respective metabolite’s median value and then converting the resulting values to log_2_. For the SNP data, genotypes were directly translated into numerical values, where the homozygous reference corresponded to 0, the heterozygous state corresponded to 0.5, and the homozygous alternate corresponded to 1. For the clustering analysis, K-means clustering was executed utilizing the open-source software Multi-experiment Viewer (MeV 4.9.0, https://sourceforge.net/projects/mev-tm4/files/mev-tm4/) [26]. The Elbow Method was used to determine the optimal number of clusters, with k = 5 being selected as the most appropriate value for both metabolomic and SNP data (Figure S2A). This analysis categorized the metabolites (Figure S2B) and the SNPs (Figure S3) into five groups based on their concentrations or genotypes and employed Euclidean distance metrics. Upon completion of the k-means clustering analysis, a heatmap was generated using MeV 4.9.0 to visually represent the clustered data.

### Machine learning (ML)-assisted prediction of CAD

When conducting the machine learning analysis, we utilized R Version 3.6.3 together with various packages, including data.Table (1.14.2), dplyr (1.1.4), stringr (1.5.0), doParallel (1.0.15), MatchIt (4.0.1), randomForest (4.6.14), e1071 (1.7.3), glmnet (4.1.4), rpart (4.1.15), xgboost (0.90.0.2), caret (6.0.85), pROC (1.16.1), and cvAUC (1.1.0). The dataset was randomly divided into training and validation sets using an 80:20 split ratio. Four ML algorithms, namely Random Forest (RF), Support Vector Machine (SVM), Least Absolute Shrinkage and Selection Operator (LASSO), and Extreme Gradient Boosting (XGBoost), were employed for the model training, feature selection, and validation. Among these algorithms, the RF model was identified as able to extract the most pertinent features while achieving the highest performance in terms of Area Under Curve (AUC) and accuracy rate. We also performed hyperparameter optimization for XGBoost, testing eta values at [0.1, 0.3, 0.5, 0.7, 0.9], max_depth at [6, 12, 29], and min_child_weight at [1, 2, 4, 8, 10], which resulted in a total of 75 parameter combinations (Table S2). However, the results of the RF and XGBoost models showed no significant difference (Table S3). Additionally, to assess the robustness of the different sampling schemes using the same selected features, we first performed feature selection and model building with 100 bootstrapped replications. For each of the 100 bootstrap iterations, the data were randomly split into training and validation sets. Three ML methods, namely LASSO, RF, and SVM, were independently applied for feature selection. In each iteration, LASSO-selected features were defined as those with non-zero coefficients; RF and SVM selected the top k features based on feature importance and weighted support vectors, respectively; where k was determined by the number of features selected by LASSO in that iteration. The selected features from all three methods were recorded, and at the end of 100 iterations, we summarized the total selection counts for each feature. Final feature ranking was determined based on the cumulative frequency of selection across all iterations and methods. The 16 top-ranked features were then used in a 10-fold cross-validation to evaluate the consistency of prediction performance, with both sampling schemes yielding similar results (Table S4). Additionally, in order to verify the stability of feature selection across the bootstrapped and cross-validation methods, we conducted an independent 10-fold cross-validation using the LASSO, SVM, and RF methods for feature selection. As shown in Table S5, 60–75% of the selected features were consistent with the bootstrapped method in each fold, and the average prediction performance was comparable to that of the bootstrapped method. In this study, we applied the RF model for the determination of model construction. The RF model was trained using an ensemble of decision trees, leveraging bootstrap sampling and random feature selection at each split to enhance robustness and reduce overfitting. Compared to other ML models, namely SVM, Decision Tree, and XGBoost, the RF model demonstrated superior performance, as shown in Figure S4.

### Statistics

The statistical analyses were conducted using SPSS software for Windows, version 25.0 (IBM Corporation, Armonk, NY). To assess the normality of the various variable distributions, the Shapiro-Wilk test was employed. For comparisons between groups based on continuous variables, either a one-way analysis of variance (ANOVA) or a Kruskal-Wallis test was utilized, depending on the nature of the data distribution. Results for continuous variables are presented as means ± standard deviations (SD) or median with an interquartile range (IQR). For the categorical variables, the distribution across groups were examined using the Freeman-Halton test, with the results expressed as frequencies and percentages. This methodology allowed for comprehensive comparisons across the various variable types in order to better understand the differences between the three groups. For the statistical analysis of SNPs, we used both χ² tests and the SAIGE [26] method. The χ² test offers a simple and computationally efficient approach for initial exploration. To account for population structure and in order to handle small *p*-values in GWAS, we employed the SAIGE method, which improves accuracy by addressing potential imbalances when there is case-control data.

## Results

### Clinical characteristics of the study’s subjects

The study encompassed 781 subjects, which were divided into a CAD group (*n* = 147), a high-risk group (*n* = 363), and a control group (*n* = 271) (Table 1). The CAD group, with a median SYNTAX score of 27.0 (IQR: 22.0–43.0), a quantitative angiographic measure of lesion complexity and disease severity [27], had a higher proportion of male participants. The CAD group and the high-risk group both exhibited a number of elevated clinical markers (age, body weight, BMI, waist circumference, SBP, DBP) compared to the control group. Comorbidities, such as diabetes and CKD, were more prevalent in the CAD group and the high-risk group. Lipid profiles (total cholesterol, HDL, low-density lipoprotein (LDL) of the CAD group was significantly lower than the other two groups, possibly due to increased statin use. The CAD group and the high-risk group showed elevated levels of TG, creatinine, adiponectin, and leptin compared to the control group. Notably, the CAD group had a higher WBC and high-sensitive C-reactive protein values than the other two cohorts.

**Table 1:** Basic characteristics of subjects in different groups

Group	Total	Control	High-risk	CAD	
n	781	271	363	147	*p*
Sex (male, %)	356 (45.6)	96 (35.4)	151 (41.6)	109 (74.1)	< 0.0001
Age (years, mean)	62.1 ± 12.7	56.3 ± 14.4	65.7 ± 10.6	63.8 ± 10.1	< 0.0001
Height (cm)	159.4 ± 9.0	158.3 ± 8.6	158.1 ± 8.8	164.3 ± 8.5	< 0.0001
Weight (Kg)	66.5 ± 13.7	59.2 ± 12.0	68.7 ± 12.2	74.6 ± 13.6	< 0.0001
BMI	26.09 ± 4.3	23.55 ± 4.06	27.37 ± 3.65	27.62 ± 4.13	< 0.0001
Waist (cm)	85.8 ± 11.8	77.2 ± 9.7	89.7 ± 9.4	94.9 ± 9.8	< 0.0001
Systolic BP (mmHg)	132.6 ± 45.7	117.6 ± 13.6	142.3 ± 62.5	136.3 ± 21.2	< 0.0001
Diastolic BP (mmHg)	76.5 ± 11	70.8 ± 9.1	80.2 ± 10.8	78.1 ± 10.7	< 0.0001
Heart rate (BMP)	75.4 ± 12.7	75.7 ± 11.2	76.2 ± 13	72.9 ± 14.5	0.028
DM (%)	249 (31.9)	2 (0.7)	156 (43.0)	91 (62.3)	< 0.0001
CKD (%)	190 (24.4)	13 (4.8)	120 (33.1)	57 (39.0)	< 0.0001
Fasting glucose (mg/dL)	111.0 ± 41.1	91.5 ± 6.6	119.8 ± 41.9	127.2 ± 60.7	< 0.0001
Glycohemoglobin (%)	6.33 ± 0.78	5.6 ± 0.31	6.5 ± 1.27	6.20 ± 1.11	< 0.0001
Total cholesterol (mg/dL)	185.8 ± 38.7	199.5 ± 31.3	186.3 ± 38.5	157.5 ± 37.2	< 0.0001
HDL (mg/dL)	54.6 ± 16.2	65.7 ± 14.5	49.6 ± 13.1	45.7 ± 14.7	< 0.0001
LDL (mg/dL)	119.6 ± 36	128.7 ± 31.1	121.4 ± 37.9	97 ± 30.1	< 0.0001
Triglyceride (mg/dL)	134.2 ± 87.3	84.8 ± 32.4	165.2 ± 100	150.4 ± 83.1	< 0.0001
BUN (mg/dL)	16.6 ± 8.5	14.2 ± 3.9	17.4 ± 7.6	19.6 ± 14.2	< 0.0001
Creatinine (mg/dL)	0.92 ± 0.63	0.74 ± 0.17	0.94 ± 0.39	1.23 ± 1.26	< 0.0001
Albumin (g/dL)	4.86 ± 8.78	4.64 ± 0.24	4.57 ± 0.26	4.53 ± 0.32	0.198
ALT (U/L)	27.55 ± 18.66	22.82 ± 11.81	29.96 ± 19.88	30.44 ± 23.84	< 0.0001
Total bilirubin (mg/dL)	0.63 ± 0.33	0.67 ± 0.29	0.6 ± 0.27	0.64 ± 0.52	0.046
Alkaline phosphatase (U/L)	71.29 ± 21.12	67.18 ± 19.29	74.95 ± 20.32	69.65 ± 25.14	< 0.0001
RBC (10^6^/µL)	4.7 ± 0.6	4.7 ± 0.55	4.78 ± 0.59	4.52 ± 0.68	< 0.0001
Hemoglobin (g/dL)	13.88 ± 1.55	13.69 ± 1.32	14.14 ± 1.52	13.56 ± 1.93	< 0.0001
WBC (10^3^/µL)	6.4 ± 2.12	5.68 ± 2.03	6.65 ± 2.01	7.14 ± 2.18	< 0.0001
Platelets (10^3^/µL)	260.7 ± 65.7	260.7 ± 59.5	266.8 ± 68.6	244.9 ± 67.3	0.004
Adiponectin (µg/mL)	8 ± 7.4	6.9 ± 5.1	7.9 ± 7.1	9.2 ± 9.5	0.105
Leptin (ng/mL)	10 ± 9.5	7.9 ± 8.4	11.5 ± 10.1	9.2 ± 8.8	0.002
HS-CRP (mg/L)	3.4 ± 13.6	2.1 ± 5.4	2.8 ± 5.8	7.5 ± 29.2	0.001
**Medications**					
Statin (%)	209 (26.8)	18 (6.6)	86 (23.7)	105 (71.4)	< 0.001
Fibrate (%)	14 (1.8)	0 (0)	10 (2.8)	4 (2.7)	0.023
CCB (%)	103 (13.2)	1 (0.4)	71 (19.6)	31 (21.1)	< 0.001
β-blocker (%)	113 (14.5)	20 (7.4)	46 (12.7)	47 (32.0)	< 0.001
ARB (%)	138 (17.7)	4 (1.5)	91 (25.1)	43 (29.3)	< 0.001
Diuretics (%)	43 (5.5)	5 (1.8)	22(6.1)	16 (10.9)	< 0.001

CAD, coronary artery disease; BP, Blood pressure; BMI, body mass index; DM, diabetes mellitus; CKD, chronic kidney disease; HDL, high-density lipoprotein; LDL, low-density lipoprotein; BUN, blood urea nitrogen; ALT, alanine aminotransferase; RBC, red blood cell; WBC, white blood cell; HS-CRP, high-sensitive C-Reactive protein; CCB, calcium channel blocker; ARB, angiotensin receptor blocker. Data are presented as the mean ± SD and analyzed by one-way ANOVA with Dunnett’s correction

### Cluster analysis of subjects according to their metabolome data

Using extensive datasets, machine learning algorithms have been able to demonstrate efficacy when identifying patterns by utilizing distance metrics to quantify dissimilarities between distinct data points [28]. For this investigation, the Euclidean distance metric was deliberately chosen to mitigate distortions within the K-means algorithm [29]. Using the Elbow Method, which indicated an optimal range of 3–5 clusters for the metabolomic data, we selected k = 5 as the most suitable value for both datasets (Figure S2A). The results revealed that, by employing the Euclidean distance metric with the designation of k = 5 subtypes, it was possible to consistently obtain higher silhouette scores and connectedness metrics, which in turn gave a normalized Jaccard similarity exceeding 0.80 for each cluster identified by the two respective methodologies.

**Fig. 2:**
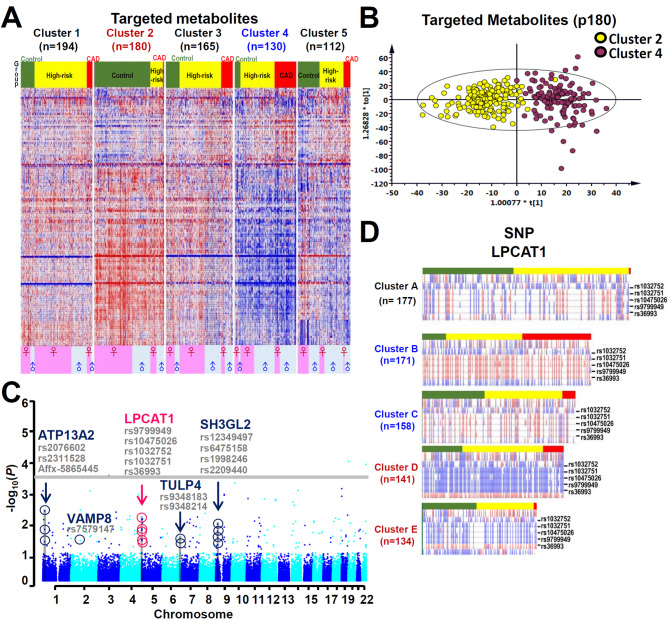
Characteristics of the subjects in different metabolomic clusters and their genetics. **A**, Clustering analysis of the dataset of 143 metabolites. Red color indicates the abundance of metabolite was up-regulated compared to the control and blue color indicates down-regulation. Each raw in the score plots represents each individual subject. The alignment of subjects, from top to bottom, was arranged from control group to high-risk group to CAD group. In each group, the female subjects were arranged on top of male subjects. In each gender, the younger subjects were arranged on top of the older ones. The colors in the heatmap reflect the plasma metabolite abundance (mean centered and divided by the range of each variable). **B**, Comparison of the 143 targeted metabolites using an orthogonal partial least squares-discriminant analysis (OPLS-DA) plot representing the separation of the plasma metabolites from cluster 2 and 4. The 143 variables and 780 observations were selected to build this OPLS-DA score plot by cross-validation rules. The sum of squares captured by this model (R2) was 0.777 and the cross-validated (Q2) was 0.705. **C**, Manhattan Plot of whole genome SNP analysis for the different groups, namely control, high-risk and CAD. Plot of negative log_10_ of the *p*-values for the single SNP association analysis of 271 control, 363 high-risk, and 147 CAD subjects; these are ordered along the x-axis for each chromosome by chromosomal position. **D**, The significant 195 SNPs (*p*-values < 0.05) are divided into homozygous reference (colored in blue), homozygous SNP (colored in red) and heterozygous SNPs (colored in white) according to their diploid genotypes. The SNPs are grouped into five clusters on the basis of Euclidean distance by K-means clustering. Each raw value in the score plots represent each individual subject. The alignment of subjects, from top to bottom, is arranged from control to high-risk to CAD group. In each group, the female subjects were arranged on top of male subjects. for each gender, the younger subjects were placed on top of older ones

All study subjects could be classified into five metabolic clusters, which are denoted as cluster 1 through cluster 5 (Fig. 2A and Figure S2B). Based on key characteristics, such as BMI, age, and gender distribution (Table S6), cluster 4 emerged as the most at-risk group, with the highest BMI (28.1), a male-dominant gender distribution (male 64.6%), and a high incidence of CAD (40%). In contrast, cluster 2, which represented the healthiest group, had a normal BMI (22.9), an average age of 59.7, and showed female dominance (female 63.3%). A boxplot depicting the distribution of these characteristics across clusters is shown in Figure S5E. Notably, cluster 4 subjects displayed the highest incidence rates of diabetes, of CKD, and various abnormal clinical indices, such as body weight and fasting glucose, among the five clusters. Conversely, cluster 2 presented with the lowest prevalence of these comorbidities and the lowest levels of TG. Interestingly, cluster 2 demonstrated the highest levels of total cholesterol and HDL, whereas cluster 4 showcased the lowest levels among the five clusters.

Table S7 outlines the 10 top-ranked metabolites that distinguish the CAD, high-risk, and control groups obtained via ML prediction. Nine of these metabolites are glycerophospholipids, while one is a sphingomyelin. The score plot, generated from an Orthogonal Partial Least Squares-Discriminant Analysis (OPLS-DA, Fig. 2B), vividly displays the distinct patterns found across the 143 plasma metabolites derived from clusters 2 and 4. These clusters exhibit marked differences in their metabolic profiles. Employing K-means cluster analysis, two distinct metabolomic patterns emerged, one of these representing the healthiest group of subjects (metabolic cluster 4), while the other represents the least healthy group of subjects (metabolic cluster 2). This analysis highlights the extreme variations present in the metabolic profiles of the subjects in this study.

### Cluster analysis of subjects according to their SNP data

To investigate potential genetic associations within the various distinct patterns of plasma metabolome, we conducted GWAS using the Axiom Genome-Wide TWB 2.0 Array. Only 195 SNPs (Figure S1A) met our stringent criteria, exhibiting adjusted *p*-values below 1 × 10^− 7^ and an Odds ratio > 1 or < 0.5 among the initial pool of 370,394 SNPs. These values were calculated using both χ² tests and the SAIGE method, and further adjusted using the Bonferroni correction. Subsequently, these selected SNPs were employed for correlation analysis focusing on the CAD group compared to the control group (Figure S1B and S1C). As shown in a Manhattan plot of the GWAS (Fig. 2C), none of the SNPs reached the genome-wide significance threshold of 2.6 × 10^− 4^ (0.05/195). Table S8 provides the genotype frequencies of these SNPs for the control, high-risk, and CAD groups.

Our examination concentrated on two distinct chromosomal regions where *p*-values were lower than 1.0 × 10^− 2^. Table S9 lists the 10 top-ranked SNPs that most effectively differentiate between the CAD group and the control group, as predicted by ML. Key SNPs, such as those in the Lysophosphatidylcholine Acyltransferase 1 (LPCAT1) gene, showed higher statistical significance using SAIGE compared to the χ² test. A comparison of *p*-values from both methods in Table S9 highlights the effectiveness of each approach in detecting associations with CAD. Additionally, the genotype frequencies of these SNPs were compared with data obtained from the Taiwan Biobank (https://taiwanview.twbiobank.org.tw/) in order to provide additional context and validation. This examination identified five genes, these being LPCAT1, SH3 Domain Containing GRB2 Like 2, Endophilin A1 (SH3GL2), Vesicle Associated Membrane Protein 8 (VAMP8), Tubby-related protein 4 (TULP4), and ATPase Cation Transporting 13A2 (ATP13A2); these genes were then extracted from the list of top-ranked SNPs. In order to pursuit further the genetic factors significantly influencing differences in metabolomic patterns across the control, high-risk and CAD groups, we unbiasedly clustered SNPs into five distinct groups (clusters A-E, Fig. 2D and Figure S3) using K-means clustering. The Elbow Method was used to determine the appropriate number of clusters, and k = 5 was selected based on its consistency with both metabolomic and SNP data clustering (Figure S2A). Comparative analysis of SNP patterns across these clusters revealed significant differences, particularly in clinical characteristics such as BMI, age, and gender distribution (Table S10). A boxplot depicting these characteristics across SNP clusters is provided in Figure S5F. For example, cluster B exhibited the highest CAD risk (47.4%), with a BMI of 26.7, and an older average age (63.6 years). On the other hand, cluster E had a low CAD risk (10.4%), with a BMI of 25.2, and an average age of 61.9 years. The comparative analysis of SNP patterns among these clusters revealed statistically significant differences. However, when we evaluated clinical characteristics such as SBP, DBP, and TG among these genetic clusters (Table S10), the observed distinctions were not as pronounced as those evident in the metabolomic clusters. Moreover, the disparity in lipid-lowering and antihypertensive medication prescriptions among the SNP clusters was not as conspicuous as observed within the metabolite clusters. This observation suggests that SNP clustering might mitigate the impact of medication on clinical manifestations. Furthermore, we analyzed the genotype frequencies of the CAD-associated SNPs that have been reported previously and that are able to be identified by the Axiom™ Genome-Wide TWB 2.0 array (Table S11). However, statistical analysis by the proportion test revealed that none of the risk alleles of previously reported SNPs pass the threshold (*p* < 0.05) for statistical significance between the control and CAD groups.

### Filtered metabolomic and genetic cluster analyses

**Fig. 3:**
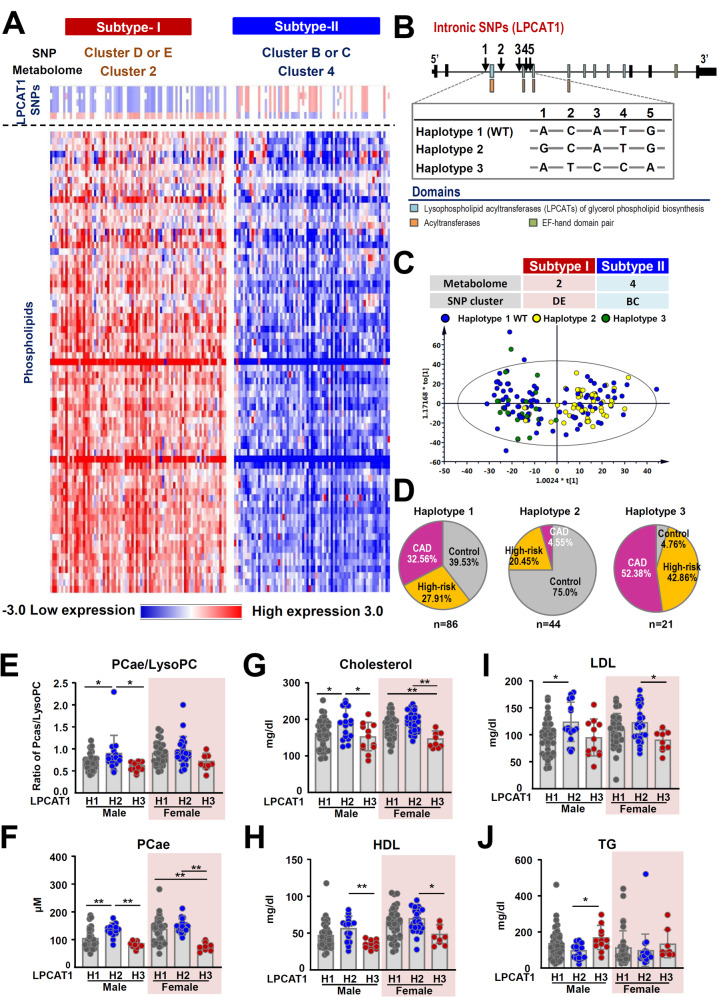
Comparisons of the subjects filtered by metabolomics and SNP cluster analyses. **A**, Bi-allelic SNPs of LPCAT1 are presented as homozygous allele 2 (SNP, red), homozygous allele 1 (WT, blue), and heterozygous SNPs (white). The pattern of glycerophospholipids detected based on targeted metabolomic abundance (mean centered and divided by the range of each variable) in each pair of subtypes of subjects aligned by SNPs are presented. **B**, Two specific haplotypes of LPCAT1 SNPs were detected as following: rs36993 (A > G), rs9799949 (C > T), rs10475026 (A > C), rs1032751 (T > C) and rs1032752 (G > A). **C**, Orthogonal partial least squares discriminant analysis (OPLS–DA) score plot of plasma metabolites of the subjects in cluster 2 and 4. Subjects with haplotype 2 or haplotype 3 have differential metabolomic patterns. In total, 143 variables and 151 observations were selected to build this OPLS-DA score plot by cross-validation rules. The sum of squares captured by this model (R2) was 0.814 and the cross-validated (Q2) was 0.755. **D**, Pie charts indicating the frequencies of the subjects’ group, filtered by metabolomic and SNP cluster analyses for the different haplotypes of LPCAT1. The haplotype 3 of LPCAT1 had a significantly increased percentage of subjects in the high-risk and CAD groups. The ratio of PCae/LysoPC **(E)** and the level of PCae **(F)** were significantly reduced in both male and female subjects with haplotype 3. In addition, the total cholesterol level **(G)**, high-density lipoprotein level **(H)** and low-density lipoprotein level **(I)** of the subjects with haplotype 3 were lower than those of haplotype 2. Furthermore, the plasma triglyceride level **(J)** of the subjects with haplotype 3 was significantly higher than the other haplotypes. Quantification data are presented as mean ± SD and analyzed by one-way ANOVA with Bonferroni multiple comparison test. *p < 0.05; **p < 0.005

Subsequent analysis revealed a prominent association between metabolomic cluster 2 and genetic clusters D and E, while metabolomic cluster 4 was correlated predominantly with genetic clusters B and C (Figure S5). Based on this association, participants within metabolomic cluster 2, which was linked to genetic clusters D/E, were grouped into “Subtype-I”, while those in metabolomic cluster 4, which was associated with genetic clusters B/C, were grouped into “Subtype-II” (Fig. 3A). It should be noted that these two subtypes exhibit significant disparities in clinical characteristics and comorbidities (Table S12). Subtype-I has notably higher total cholesterol, HDL, and LDL levels compared to Subtype-II. Moreover, the latter subtype exhibited a higher representation of individuals from the high-risk and CAD groups. Pronounced variations in specific metabolomic markers (particularly glycerophospholipids) and genetic markers (specifically LPCAT1) were observed when the two subtypes were compared (Figs. 2D and 3A, and Figure S6). To validate these associations, we performed the Chi-square test and compared the observed SNP distributions in each of the metabolomic cluster. SNP clusters D and E were significantly enriched in metabolomic cluster 2 (*p* = 2.5 × 10⁻¹⁹), while SNP clusters B and C were overrepresented in metabolomic cluster 4 (*p* = 1.13 × 10⁻¹³). These results confirm that the clustering patterns are statistically significant and supporting the hypothesis that there is a genetic-metabolic association based on our finding that there is a connection between the genomic and metabolomic datasets (Figure S5B). Additionally, analysis of the genotype frequencies of the five LPCAT1 SNPs (rs1032752, rs1032751, rs10475026, rs9799949 and rs36993) across the control, high-risk, and CAD groups revealed a significant difference (*p* = 0.0365) by ANOVA. Notably, the proportion test showed a significant difference between the control and CAD groups for these LPCAT1 SNPs (*p* = 0.0295), which suggests that there is potential linkage disequilibrium as well as a possible haplotype linkage; this might be associated with a genetic predisposition towards CAD (Figure S7).

Further investigation revealed two distinct haplotypes of LPCAT1 associated with different metabolomic clusters. Haplotype 2 was defined by a specific combination of homozygous references, namely rs1032752, rs1032751, rs10475026, rs9799949, along with a homozygous SNP, rs36993. Conversely, haplotype 3 was characterized by homozygous SNPs, rs1032752, rs1032751, rs10475026, rs9799949, paired with a homozygous SNP, rs36993. All other combinations were classified as haplotype 1 (wild type, WT; Fig. 3B). Examining the impact of the LPCAT1 haplotypes on plasma metabolomic patterns using OPLS-DA (Fig. 3C) indicated that subjects with haplotype 2 or haplotype 3 have differential metabolomic patterns. These findings suggest a significant influence of specific LPCAT1 haplotypes on a subject’s plasma metabolomic expression.

### The LPCAT1 haplotypes affect the phosphatidylcholine profile

LPCAT1 influences disease progression by enhancing the level of saturated phosphatidylcholine (PC) [30]. Notably, a higher percentage of individuals with the LPCAT1 haplotype 3 were found in the high-risk (42.86%) and CAD (52.38%) groups, while LPCAT1 haplotype 2 individuals showed a lower association with the high-risk group (20.45%) and the CAD groups (4.55%) (Fig. 3D). LPCAT1 haplotype 1 (WT) was found to be evenly distributed across all three groups. When a gender-specific analysis was carried out, male participants with the LPCAT1 haplotype 2 displayed an increased PCae/LysoPC ratio (Fig. 3E), a higher PCae level (Fig. 3F), a higher total cholesterol level (Fig. 3G), a higher HDL level (Fig. 3H), and a lower TG level (Fig. 3J) compared to haplotype 3. Conversely, females with the LPCAT1 haplotype 3 exhibited a lower PCae level (Fig. 3F), a lower total cholesterol level (Fig. 3G), a lower HDL level (Fig. 3H), and a lower LDL level (Fig. 3I) levels compared to haplotype 2. These findings indicate there are gender-specific differences in various lipid profiles and these are associated with LPCAT1 haplotypes within the groups.

### The ML-assisted identification of CAD risk factors across the whole cohort

We extended our investigation to explore the potential role of the LPCAT1 haplotypes in influencing phenotypic and metabolomic patterns across the entire cohort. This used an integrated ML-assisted analysis to identify specific risk factors among the control, high-risk, and CAD groups; thus leveraging the previously mentioned data [20]. This comprehensive study comprised three main segments: (1) conducting GWAS using the Axiom Genome-Wide TWB 2.0 Array; (2) selecting metabolomic and clinical characteristic features; and (3) deriving and validating predictive models. Among various machine-learning models, the Random Forest model was found to show superior performance in terms of AUC and accuracy rate (Figure S4).

**Fig. 4:**
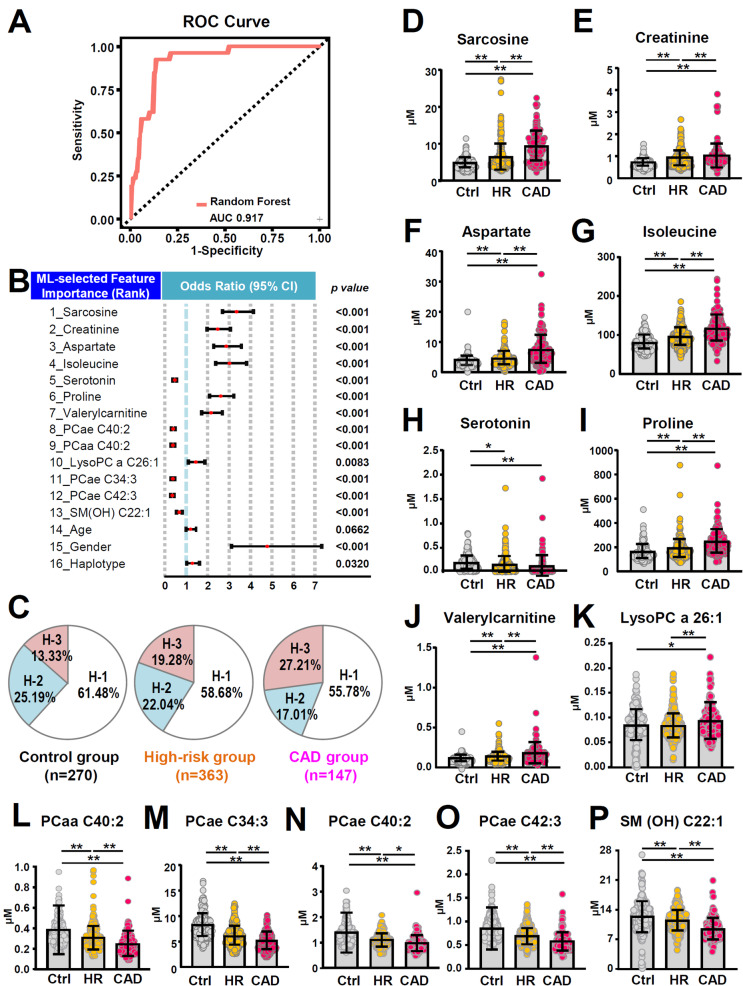
ML-assisted prediction of coronary artery disease (CAD). A ML-assisted trans-omics prediction model, based on clinical features, metabolome, and genome-wide SNPs, was used to identify patients with CAD across the whole cohort. **A**, The receiver operating characteristic (ROC) curve for predicting CAD. **B**, Adjusted odds ratios of features associated with CAD patients. The risks were estimated by the backward stepwise selection method. **C**, The frequency of LPCAT1 haplotype-3 is increased in the high-risk group and then is further raised in the CAD group. **D-I**, The deregulated levels of various clinical risk factors, amino acids and biogenic amines associated with CAD subjects, including sarcosine **(D)**, creatinine **(E)**, aspartate **(F)**, isoleucine **(G)**, proline **(H)** and serotonin **(I)**. **J-P**, The lipidomic pattern, with an elevation of valerylcarnitine (**J**) and lysoPC a26:1(**K**), as well as a decrease of other lipids, including PCaa C40:2 (**L**), PCae C34:3 (**M**), PCae C40:2 (**N**), PCae C42:3 (**O**), and SM(OH) C22:1 (**P**); these were associated with progression from the control group to the high-risk group to the CAD group. Quantification data are presented as mean ± SD and analyzed by one-way ANOVA with Bonferroni multiple comparison test. AUC, area under the curve. *p < 0.05; **p < 0.005

Figure 4A illustrates the ML-assisted CAD prediction performance across the entire cohort by utilizing the 16 top-ranked interpretable features outlined in Fig. 4B; this achieved an accuracy rate of 0.805 and an AUC of 0.917. Interestingly, the distribution of individuals carrying the LPCAT1 haplotype 1 (H-1) across the control, high-risk, and CAD groups was similar (Fig. 4C). However, a notable increase in the percentage of participants carrying the LPCAT1 haplotype 3 (H-3) could be observed across the three groups, this rose from 13.33% in controls, to 19.28% in the high-risk group, and then to 27.21% in the CAD group (χ^2^ of *p* = 0.02). In addition to the LPCAT1 haplotype, 15 other risk factors were identified. Elevated levels of sarcosine, creatinine, aspartate, isoleucine, proline, valerylcarnitine, LysoPCa C26:1, and having a male gender were all positively associated with the CAD group. Conversely, lower levels of serotonin, PCaa C40:2, PCae C34:3, PCae C40:2, PCae C42:3, and SM(OH) C22:1 were linked to the CAD group (Fig. 4D and P). When distinguishing between the high-risk and CAD groups, the ML-assisted prediction model achieved an accuracy rate of 0.740 and an AUC of 0.880 on differentiating between these two groups (Table S13). Although the performance was slightly decreased compared with the prediction of CAD using full datasets that include control, high-risk and CAD (Fig. 4A), the AUC, a measurement of sensitivity and specificity, remained at a high score of 0.88. These findings underscored that there are distinct associations between CAD and various metabolites as well as various clinical factors within the cohort.

## Discussion

This study identifies several pivotal findings regarding CAD. **Firstly**, it shows that decreased plasma phospholipids are associated with high-risk patients and that there are further declines observed in CAD patients. This progressive decrease in phospholipids across control, high-risk, and CAD groups suggests a potential role for these lipids in the mechanism of CAD progression and as biomarker for CAD progression. **Secondly**, among the 10 top-ranked SNPs linked to CAD, five genes were identified as contributors to phosphatidylcholine pattern alterations. **Thirdly**, on employing a trans-omics approach, we identified a specific LPCAT1 haplotype to be associated with CAD patients. **Lastly**, using ML-assisted techniques, we established a trans-omics predictive model for CAD and confirmed that LPCAT1 haplotype is one of the 16 top-ranked risk factors in relation to CAD prediction. Overall, our findings highlight a multi-omics signature for CAD, and this, in turn, suggests that disturbances in glycerophospholipid metabolism due to LPCAT1 genetic variants seem to be associated with the occurrence of CAD.

### The significance of the trans-omics approach

Despite numerous genetic loci having been linked to CAD via genome-wide association studies, a mere 5% of predicted CAD-related SNPs are situated within gene boundaries plus the 30 kilobase pairs at either end of a gene [30]. Most of these loci demonstrate modest effects on a range of common diseases, making them challenging to use experimentally. Consequently, these associations offer limited mechanistic insights and have little in the way of translational applications. In this context, understanding allele interactions remains critical. Utilizing a trans-omics approach with parallel measurements across multiple omics is able to offer a more comprehensive comparison, better validation, and significant enhancement compared to existing integration methods [7]. However, the application of the trans-omics approach to CAD studies has been relatively limited up to the present [31]. Delineated metabolomic patterns in CAD offers clues that allow physiological and pathological exploration of the disease [32, 33]. Nonetheless, further exploration is needed in order to elucidate the relationship between plasma metabolic profiling and the detailed characterization and quantification of metabolomic profiles in high-risk and CAD patients. Compared to the large-scale GWAS reported by Aragam et al. [5], which focused on European populations, our study takes a multi-omics approach by integrating SNP and metabolomic data from a Taiwanese cohort. While Aragam et al. found no significant associations for LPCAT1, our metabolomics data suggest a possible role for this gene in CAD within the Taiwanese population, which points to there being potential population-specific genetic variation.

### Metabolic pattern transition from high-risk patient to CAD patient

Plasma metabolites are postulated to serve as markers and effectors in cardiometabolic diseases [32]. Inter-individual metabolite variation may result from heritable factors, such as genome-wide significant loci, and clinical factors, such as sex, age, medication, comorbidities, and environmental factors. Approximately 20% of the variation in 66% of plasma metabolites is estimated to stem from heritability, while less than 20% of the variation in 93% of plasma metabolites is due to clinical factors [32]. Our study design involved three participant groups instead of two. This structure was aimed at eliminating confounding clinical factors and focused on potential heritable factors that are associated with CAD. Notably, the elevated levels of three amino acids (aspartate, isoleucine, proline) in the high-risk and CAD group participants suggest that these are associated with CAD risk factors such as type II diabetes mellitus and chronic inflammation. Additionally, an increased sarcosine level, a metabolite derived from dietary choline, was found to be associated with CAD, adding to the links between choline-derived metabolites and adverse cardiovascular events.

In plasma, amino acids and lysophosphatidylcholine (LysoPC) show considerable heritability [34]. Literature reports have highlighted altered plasma metabolomic profiles in individuals with CAD, including disruptions to aspartate and glutamate homeostasis and increased levels of isoleucine [35, 36]. Our study also detected heightened plasma levels of aspartate and isoleucine in our high-risk participants, and, notably, these were even more elevated in individuals who had been diagnosed with CAD. Furthermore, elevated proline, another amino acid identified in our findings, was found to be associated with type II diabetes mellitus and chronic inflammation, which are established risk factors for CAD. Chronic sterile inflammation serves as a connection between type II diabetes mellitus and CAD [37], as evidenced by significantly elevated levels of high-sensitive C-reactive protein (HS-CRP) in the CAD group compared to the control and high-risk groups (Table 1). This suggests a potential link between inflammation and CAD. Although elevated serotonin has been associated with coronary microvascular disease and coronary artery disease, the wide range in plasma cutoff levels used for diagnosis produces significant variation [38, 39]. Interestingly, our results revealed decreased plasma serotonin levels in the high-risk and CAD group participants.

Our analysis identified key genetic and metabolic differences between the high-risk and CAD groups. As shown in Fig. 4, the frequency of LPCAT1 haplotype 3 increased progressively from 13.33% (control) to 19.28% (high-risk) and 27.21% (CAD) (χ², *p* = 0.02), which suggests that it has a potential role in disease progression. Additionally, distinct metabolomic shifts were observed when CAD was present, such as elevated levels of valerylcarnitine and LysoPCa C26:1, as well as decrease in the PCaa and PCae subclasses; these changes serve to further differentiate these two groups. Moreover, elevated plasma sarcosine, a downstream metabolite of dietary choline, showed an association with CAD. Metabolites derived from choline produced by gut microbiota, such as trimethylamine and trimethylamine-N-oxide, have been linked to increased cardiovascular risk [40]. The metabolic pathway of dietary choline, which progresses via glycine, betaine and dimethylglycine and culminates in sarcosine, in parallel with a decrease in glycine betaine and an increase in dimethylglycine, has been linked to cardiometabolic risks and atherosclerosis [41, 42]. Our results provide additional evidence supporting the association between sarcosine level and CAD.

### Phosphatidylcholine and lysophosphatidylcholine in CAD

Phospholipids, primarily phosphatidylcholine and sphingomyelin, serve as crucial constituents of the plasma membrane and are the binding sites for proteins within the plasma membrane [43]; these are synthesized in the liver and subsequently secreted into the bloodstream. They have been implicated in glucose metabolism, with low levels of these metabolites correlating with insulin resistance [44] and with an elevated risk of Type 2 diabetes mellitus [45]. Minute alterations in phospholipid levels across various tissues can have a significant impact on the parameters associated with metabolic syndrome, such as lipid profile, obesity, and insulin resistance.

Phosphatidylcholines encompassing a single fatty acid are termed LysoPC. These lipid compounds, which act as secondary messengers, are linked to various metabolic disorders, insulin resistance, and obesity [46]. Their signaling cascade operates through the G protein-coupled receptor G2A and various Toll-like receptors [47]. PC, when metabolized by gut microbiota, has been shown to promote cardiovascular disease [48]. PCs and sphingomyelin within HDL are presumed to play a protective role against atherosclerosis. Perturbations in these lipids can impair HDL function, potentially leading to arteriosclerosis [49].

Among plasma metabolites, specific phosphatidylcholines, notably PC(32:1), PC(33:0), PC(35:1), PC(36:1), and PC(36:3), exhibit an inverse relationship with the coronary artery calcium score [50]; furthermore, studies have associated lower levels of PCs with cardiovascular disease [51]. Previous study revealed that PC (a substrate) can be hydrolyzed by the enzyme phospholipase to release fatty acid and LysoPC (a product) [52]. Interestingly, a trend towards higher levels of certain LysoPCs was found by other group [51] and this study (Fig. 4K). This is consistent with the metabolism of PC, namely there is a decrease in PC (Fig. 4L-O) that is accompanied by an increase in LysoPC (Fig. 4K). In addition, elevated fasting plasma valerylcarnitine has been linked to increased risk of cardiovascular death and of myocardial infarction among individuals with stable angina [53]. Valerylcarnitine, derived from branched-chain amino acids or odd carbon-chain fatty acid metabolism, may serve as a signature for insulin resistance in CAD participants [54, 55]. Our results demonstrated significantly higher plasma levels of valerylcarnitine and LysoPC 26:1 in CAD participants compared to the high-risk and control groups. Intriguingly, lower plasma levels of PCae were found to be associated with the CAD group of subjects compared to the other two groups.

### Lipid profiles are associated with SNPs

**Fig. 5:**
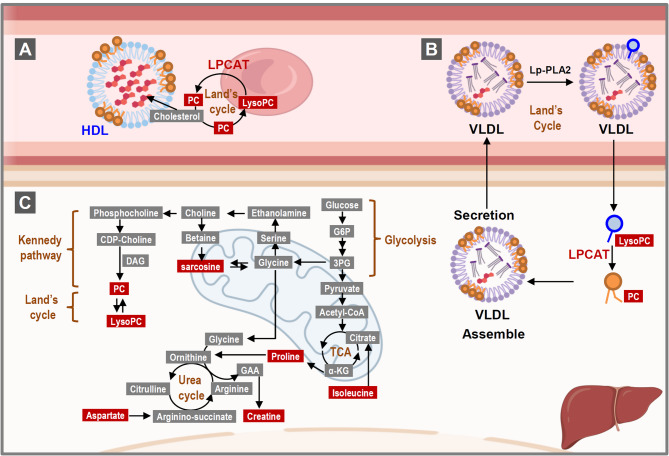
Graphic summary illustrating the trans-omics signatures of CAD patients. **A**, In the Land’s cycle, lysophosphatidylcholine acyltransferase (LPCAT) reacts with phosphatidyl coenzyme A and transforms LysoPCs into PCs. LysoPC is increasingly recognized as a key factor that is positively associated with atherosclerosis. **B**, LPCAT1 has also been found to be localized in the plasma membrane, endoplasmic reticulum, and the membrane of lipid droplets. Intracellular LPCAT rapidly converts LysoPCs into PCs, which are then assembled and secreted as VLDL. However, elevated intracellular LysoPCs will increase the amount of LysoPCs in the LDL and oxidated LDL and this results in atherosclerosis. **C**, The disturbed interaction of the Land’s cycle and the Kennedy pathway might further affect the metabolism of choline resulting in increased sarcosine in the CAD patients. Elevated sarcosine might affect the downstream TCA and urea cycles. Abbreviations: 3PG, 3-phosphoglycerate; αKG, α-ketoglutaric acid; CDP-choline, cytidinediphosphocholine; DAG, diacylglycerol; GAA, guanidinoacetic acid; G6P, glucose 6-phosphate; HDL, high-density lipoprotein; LysoPC, Lysophosphatidylcholine; LPCAT, lysophosphatidylcholine acyltransferase; Lp-PLA2, lipoprotein-associated phospholipase A2; PC, phosphatidylcholine; TCA, tricarboxylic acid; VLDL, very low density lipoprotein

LPCAT1 plays a crucial role in the Lands’ circulation by transforming lysoPCs into PCs using phosphatidyl coenzyme A (Fig. 5) [56]. LPCAT1 has been found to be localized on the plasma membrane, in endoplasmic reticulum, and in lipid droplet membranes [57, 58]. Its action as an enzyme brings about PC re-acylation in alveolar type II cells and red blood cells, and thus it regulates inflammatory lipid levels, and mediates O-palmitoylation of histone H4 in the nuclei [59]. LPCAT1, which is under cellular redox status control and Ca^2+^ concentration control, is able to re-acylate lysoPCs into PCs, thus removing oxidized and damaged unsaturated fatty acids from glycerophospholipids [57]. Dysregulation of LPCAT1 has been linked to cancer cell genetic alterations, abnormal metabolism, plasma membrane restructuring, and malignant transformations with a poor prognosis [27]. Loss of LPCAT1 in cells exposed to excessive polyunsaturated fatty acids exacerbates cytotoxicity and induces various cellular responses via pathways including the unfolded protein response [60]. Moreover, recent studies have further implicated LPCAT1 in myocardial infarction, as evidenced by the findings from GWAS and other genetic datasets available in the dbGAP Gene-Trait Associations database. These investigations have suggested a potential association between LPCAT1 and increased susceptibility to myocardial infarction, underscoring its significance beyond its known roles in lipid metabolism and inflammation [61].

In our findings, we observed an association between LPCAT1 haplotype 3 and CAD, which points to there being a significant impact of an individual’s LPCAT1 haplotype on their plasma lipidomic pattern. Additionally, other identified SNPs, such as ATP13A2 and VAMP8, are also involved in lipid metabolism. ATP13A2 dysfunction results in lysosomal dysfunction, oxidative stress susceptibility, impaired mitochondrial function, and cell toxicity [62]. Furthermore, ATP13A2 overexpression leads to increased intracellular phosphatidylethanolamine [63]. VAMP8 is involved in membrane vesicular trafficking and shares a molecular pathway that has been associated with both CAD and periodontitis. These findings collectively highlight the interplay between various genetic variants associated with lipid metabolism and pinpoints their potential role in cardiovascular disease mechanisms [64].

### Limitations and perspectives

Despite the careful utilization of clustering methodologies to explore extensive multi-omics datasets, which encompass clinical profiles, targeted metabolomics (p180-metabolites), and SNP information, in order to obtain insights into their associations with CAD, several areas warrant attention as limitations to our study. **Firstly**, our focus is primarily on targeted circulating metabolites when carrying out the CAD association, and this leaves open the question as to whether other metabolites might also influence our CAD prediction models. This question remains unanswered. **Secondly**, the unbalanced demographics between groups may have complicated our data interpretation. Key limitations include demographic differences, such as age, gender and comorbid conditions, all of which can introduce bias. The study’s observational nature limits causality, identifying associations rather than definitive relationships. Additionally, uncontrolled confounding factors, such as lifestyle and environmental influences, might have also affected our findings. The lack of imaging-based examinations in the high-risk group means that underlying subclinical atherosclerosis and/or undiagnosed CAD cannot be ruled out. A case-control study design that matches key variables would simplify interpretation and would provide clearer insights. **Thirdly**, design of our study, which is a cross-sectional one and derived from a single ethnic group, may restrict the generalizability of our findings to other populations. Thus, validation using a larger and more diverse cohort that includes ethnic diversity is essential. **Fourthly**, while we have identified a LPCAT1 haplotype as having a potential functional influence on LPCAT1 activity, its precise functional implications remain unknown. This will necessitate further investigation involving in vitro and in vivo models. **Lastly**, in order for the multi-omics signature to evolve into a biomarker profile for personalized care of CAD patients, a longitudinal study validating the utility of our prediction model over time is needed.

Regarding the imbalanced issue wherein most of the participants in this study do not have CAD, we carried out a balance test using different prediction models by subsampling the training datasets with the ratio of CAD to non-CAD set at 1:1 (Table S14). Our results revealed that the sensitivity, specificity, accuracy and AUC were similar to the original results (Table S3). However, when we match age and gender during the sampling process, the accuracy and AUC drop by about 5% (Table S14). Other performance matrices, including AUC-precision recall curve (AUC-PR), Brier score and calibration curve [65], are presented in Figure S8. We achieved an AUC of 0.92 and an AUC-PR of 0.74. AUC-PR may be sensitive to precision (PPV, Positive Predictive Value); however, precision is dependent on disease rate and it will change when disease rate is different. Regarding the Brier score and the calibration curve, these are more suitable when targeting a risk assessment application (more like a regression issue). The calibration curve is used to compare the model’s predicted probabilities and verify whether it is consistent with the observed probabilities. However, our model’s predicted probabilities tended to be higher than observed probabilities. Therefore, we adapted a probability calibration method. The slope of the calibration curve becomes closer to 1 after adaption, but this caused a lower AUC. Since our model is dealing with a classification issue and trying to identify important features related to potential pathogenetic factors associated with disease progression, we have focused in this study on a model that provides a higher AUC.

To understand the potential influence of medication on our study, we conducted the following experiments. Firstly, six medications (statin, fibrate, β-blocker, calcium channel blocker, angiotensin receptor blocker, and diuretics) were included in model analysis; to do this we used matched sampling to ensure that the same usage proportion in the CAD and non-CAD groups. Compared to the matched sampling without medication adjustment, we found that matching by age, gender, and statin slightly decreased the model performance (Table S15). Secondly, we created new features based on the assumption of an interaction between statin and metabolites, and carried out the model analyses again. Notably, when matching the statin condition, there was a big drop in the specificity (it drops from 0.849 to 0.526), indicating that adding interaction features into the model seemed to result in a misclassification of non-CAD subjects as CAD patients (Table S16).

## Conclusion

Our findings underscore the potential of integrating machine learning models with traditional approaches in order to uncover new biological mechanisms. When we compared our approach to various existing risk assessment tools (Table S17) in order to identify individuals at high risk of CAD, our prediction model showed promise. Our approach appears to outperform many demographic-based, blood biochemistry-based, and image-based assessment methods; nevertheless, further validation is necessary. An understanding of the key SNPs associated with CAD and their interactions with metabolites linked to CAD will shed light on the potential genetic predispositions and metabolic changes that form the complex disease CAD. Ultimately, the creation of a multi-omics predictive model that is capable of distinguishing CAD patients from high-risk individuals will present an avenue for developing personalized medical strategies for CAD prevention.

## Supplementary Information

Below is the link to the electronic supplementary material.

Supplementary Material 1

## Data Availability

The SNP datasets generated and analyzed in this study are available in the Gene Expression Omnibus (GEO) repository; accession code GSE303414. In addition, both the SNP and metabolomics datasets can be downloaded directly from NHRI website: http://bioinfolab.nhri.edu.tw/pub/bio2025.html.

## Abbreviations

ANOVA: Analysis of variance.
ASCVD: Atherosclerotic cardiovascular disease
AUC: Area under the curve
AUC-PR: Area under the curve-precision recall curve
BMI: Body mass index
CAD: Coronary artery disease
CKD: Chronic kidney disease
DBP: Diastolic blood pressure
GWAS: Genome-wide association studies
HDL: High-density lipoprotein
LASSO: Least absolute shrinkage and selection operator
LDL: Low-density lipoprotein
LPCAT1: Lysophosphatidylcholine acyltransferase 1
ML: Machine learning
OPLS-DA: Orthogonal partial least squares-discriminant analysis
SBP: Systolic blood pressure
SNPs: Single-nucleotide polymorphisms
SD: Standard deviations
SVM: Support vector machine
TG: Triglyceride
XGBoost: Extreme gradient boosting
