# Supplementary material for: Trans-omics analyses identify the biochemical network of LPCAT1 associated with coronary artery disease
Source: Biomark Res. 2025 Aug 20;13:107. doi: 10.1186/s40364-025-00821-y (PMC12366056; doi:10.1186/s40364-025-00821-y)
Supplement: Supplementary file 1 — Supplementary Material 1 [file 40364_2025_821_MOESM1_ESM.pdf]

Figure S1

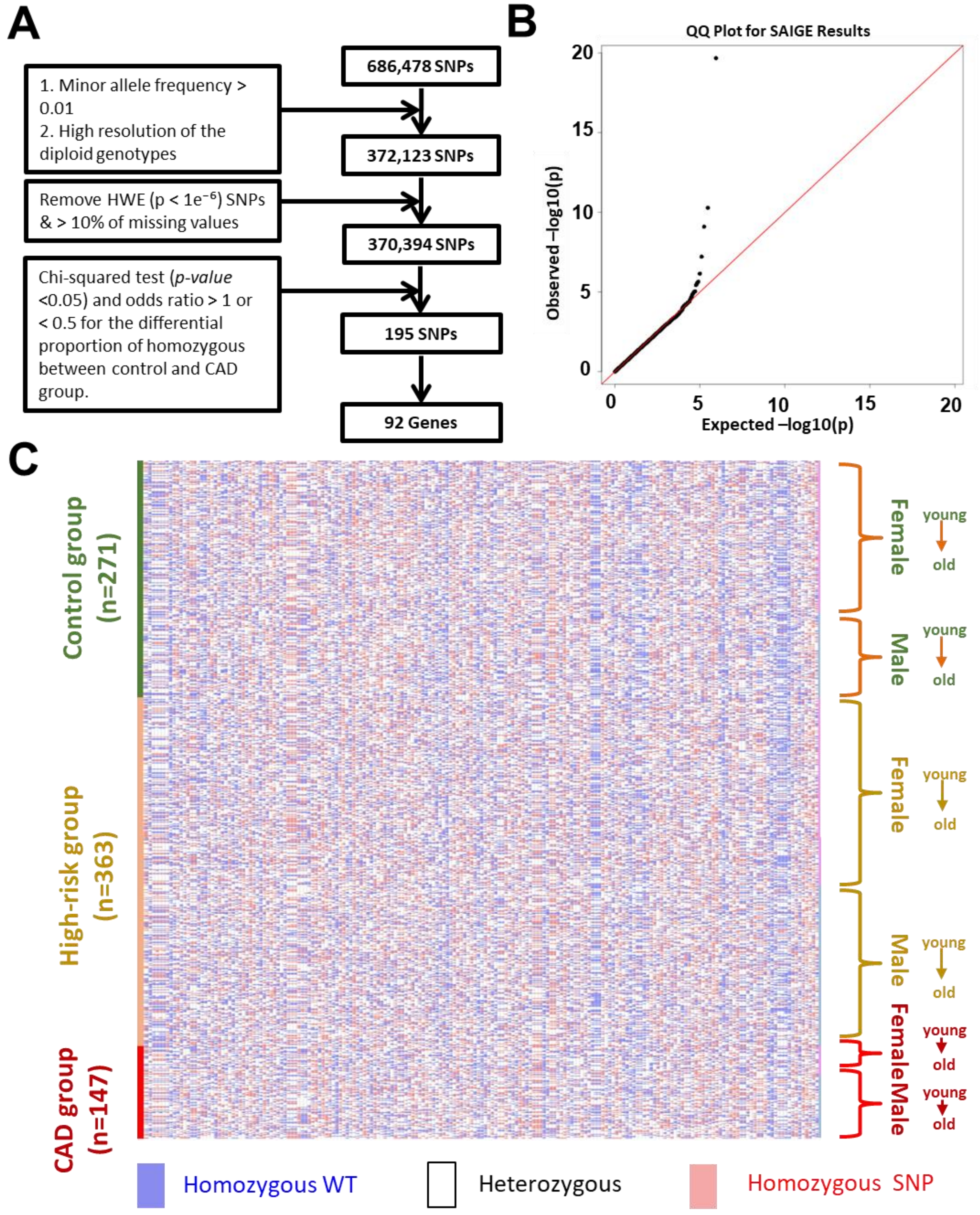

Figure S1. Overview of the 195 SNPs analysis from all subjects. (A) Flowchart of SNP selection. 686,478 SNPs were included in the AxiomTM Genome-Wide TWB 2.0 array plates. Among the SNPs identified, those with a minor allele frequency < 0.01, deviation from Hardy-Weinberg equilibrium (HWE) in controls ($p < 1\times10^{-6}$) and SNPs with a missing rate of more than 10% were excluded from further analysis. In total, 370,394 SNPs were available for further analyses. To compare the difference between CAD and control group, only those SNPs with a significant Chi-square test (p<0.05) and odds ratio >1 or <0.5 were selected for further analysis. **(B)** The quantile-quantile (QQ) plot compares observed and expected p-values for SNPs, based on SAIGE results. **(C)** The heatmap of the 195 selected SNPs.

Figure S2

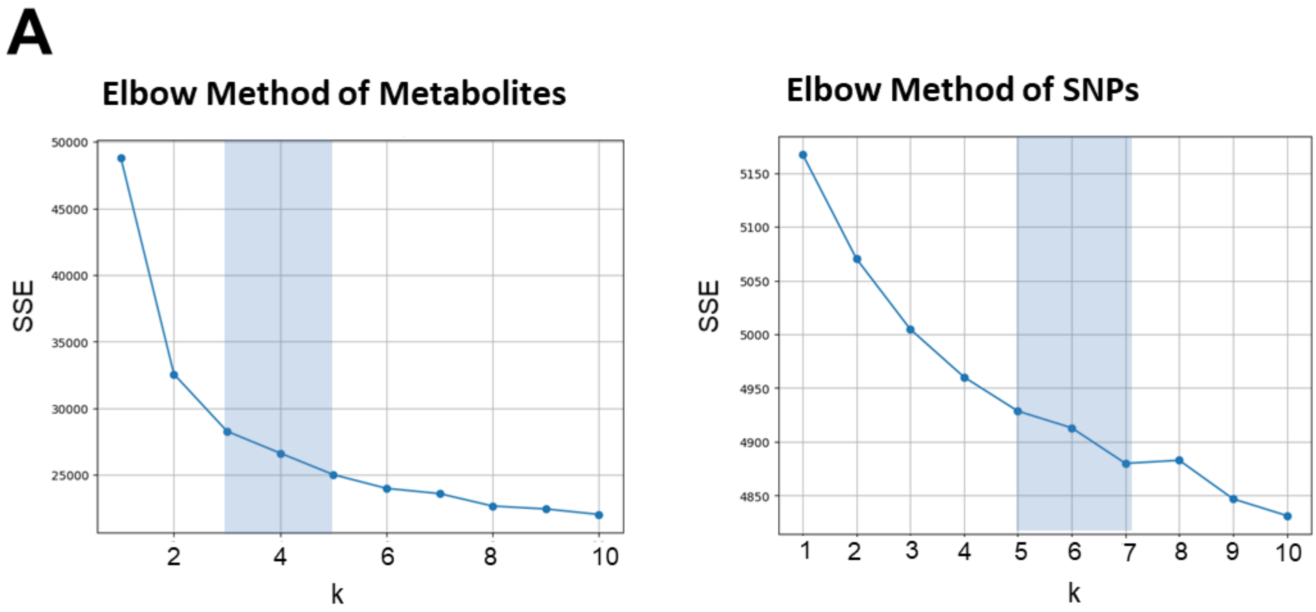

Figure S2. (A) Determination of optimal cluster number using the Elbow Method. The Elbow Method identified 3-5 clusters for metabolomic data and 5-7 for SNP data. We selected k=5 as a consistent choice for both datasets, as it marks the point where the reduction in within-cluster variance slows ("elbow"). **(B) Characteristics of subjects in different metabolomic clusters.** Clustering analysis of the 143 metabolomic data. Red color indicates the abundance of metabolite were up-regulated compared to that of the control and blue color indicates down-regulation. Each raw in the score plots represent each individual subject. The alignment of subjects, from top to bottom, was arranged from control to high-risk to CAD group. In each group, the female subjects were arranged on top of male subjects. In each gender, the younger subjects were on top of older ones. The colors in the heatmap reflect the plasma metabolite abundance (mean cantered and Divided by the range of each variable).

Figure S2 continued

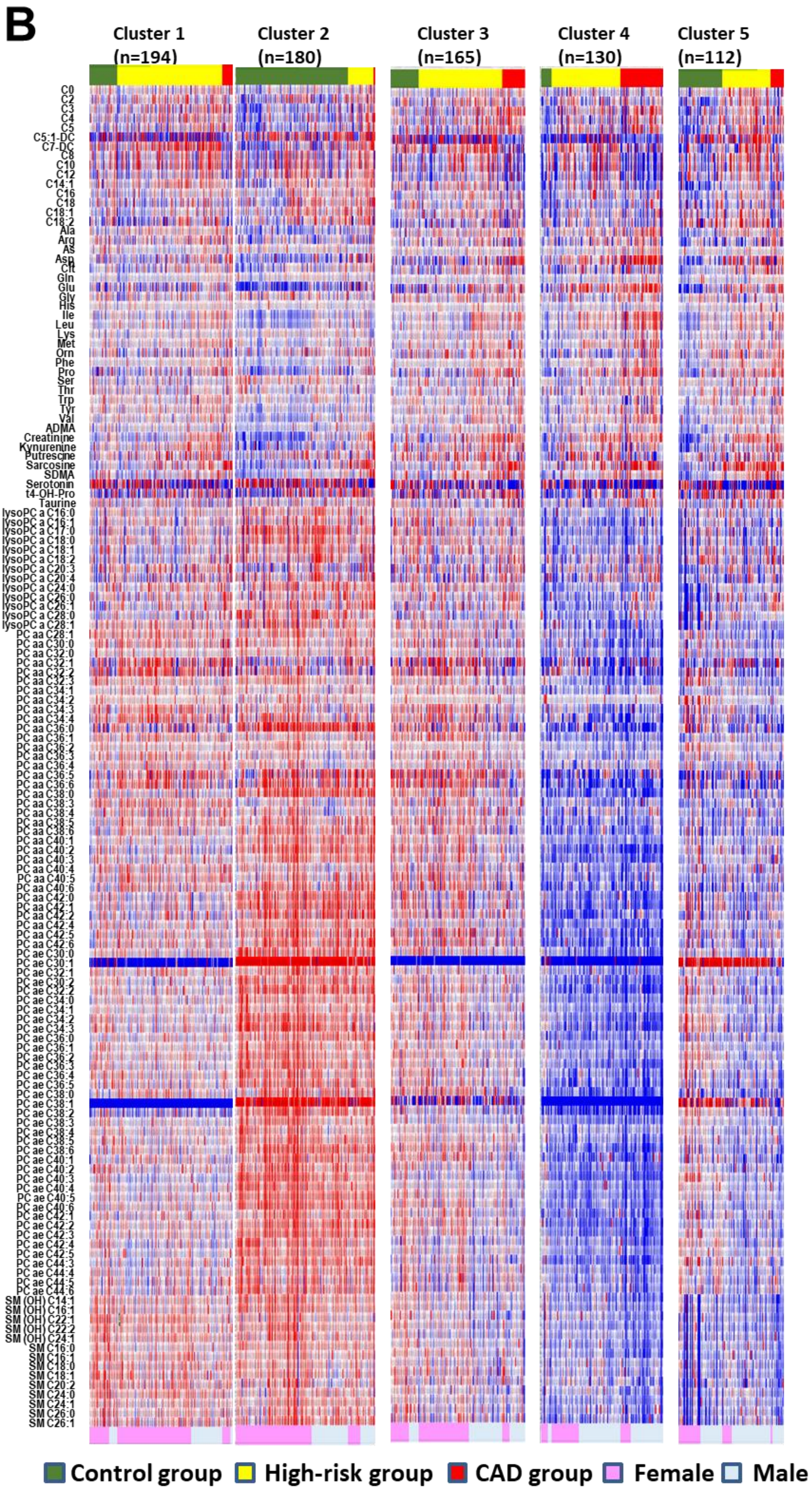

Figure S3

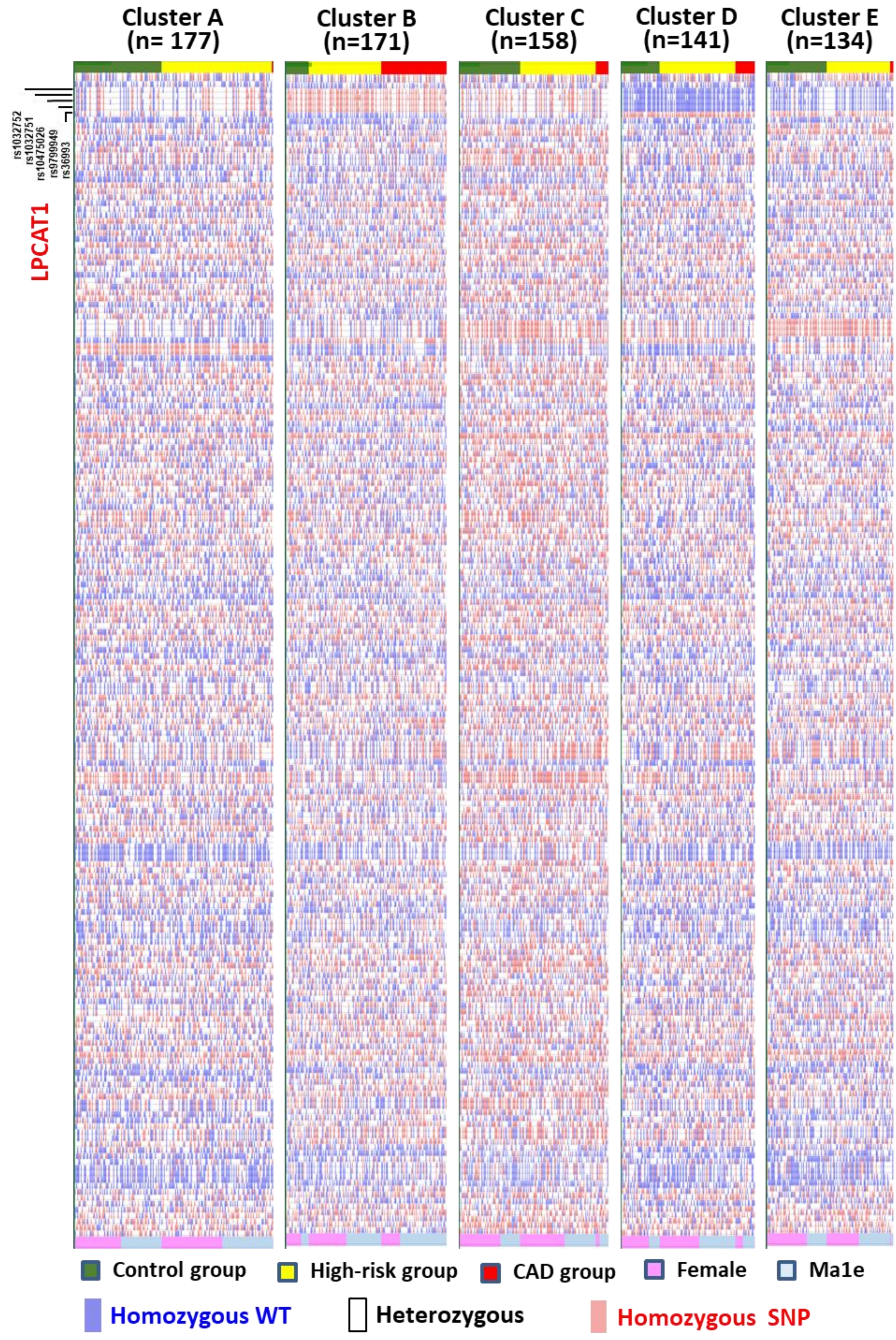

Figure S3. Overview of 195 SNPs analysis from all subjects.

Figure S4

A

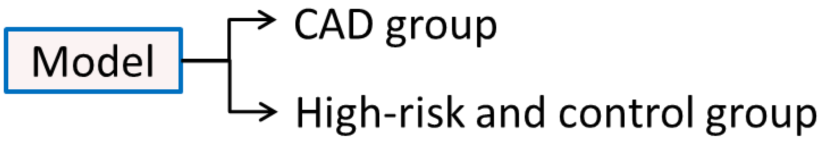

Cross-validation of prediction performance in different models

Method	Sensitivity	Specificity	PPV	NPV	Accuracy	AUC
RF	0.877(0.054)	0.790(0.042)	0.477(0.053)	0.968(0.013)	0.805(0.034)	0.917(0.023)
SVM	0.853(0.066)	0.783(0.040)	0.461(0.046)	0.962(0.016)	0.795(0.031)	0.901(0.025)
DT	0.816(0.103)	0.757(0.063)	0.425(0.055)	0.952(0.024)	0.767(0.046)	0.831(0.046)
XGB	0.836(0.072)	0.796(0.043)	0.472(0.057)	0.958(0.017)	0.803(0.035)	0.902(0.027)

B

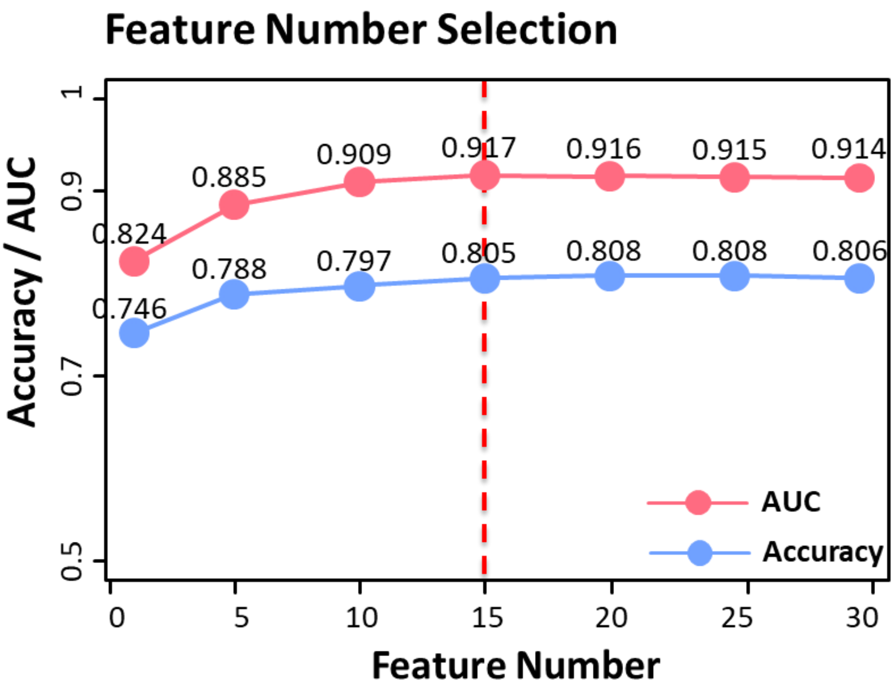

Figure S4. Prediction models built based on the clinical features, metabolomic features, and genome-wide SNPs were used to identify CAD patients using machine learning methods. (A) Machine learning workflow to select CAD patients. Classification performance of the four supervised machine learning algorithms, namely Random Forest (RF), Support Vector Machine (SVM), Decision tree (DT), and XGBoost (XGB), with selected feature from multi-omics datasets with the input dataset having a train-to-validation split ratio of 80:20. **(B)** The Accuracy and AUC of the RF model with different feature selection numbers. PPV, positive predictive value; NPV, negative predictive value; AUC, area under the curve of receiver operating characteristic.

Figure S5

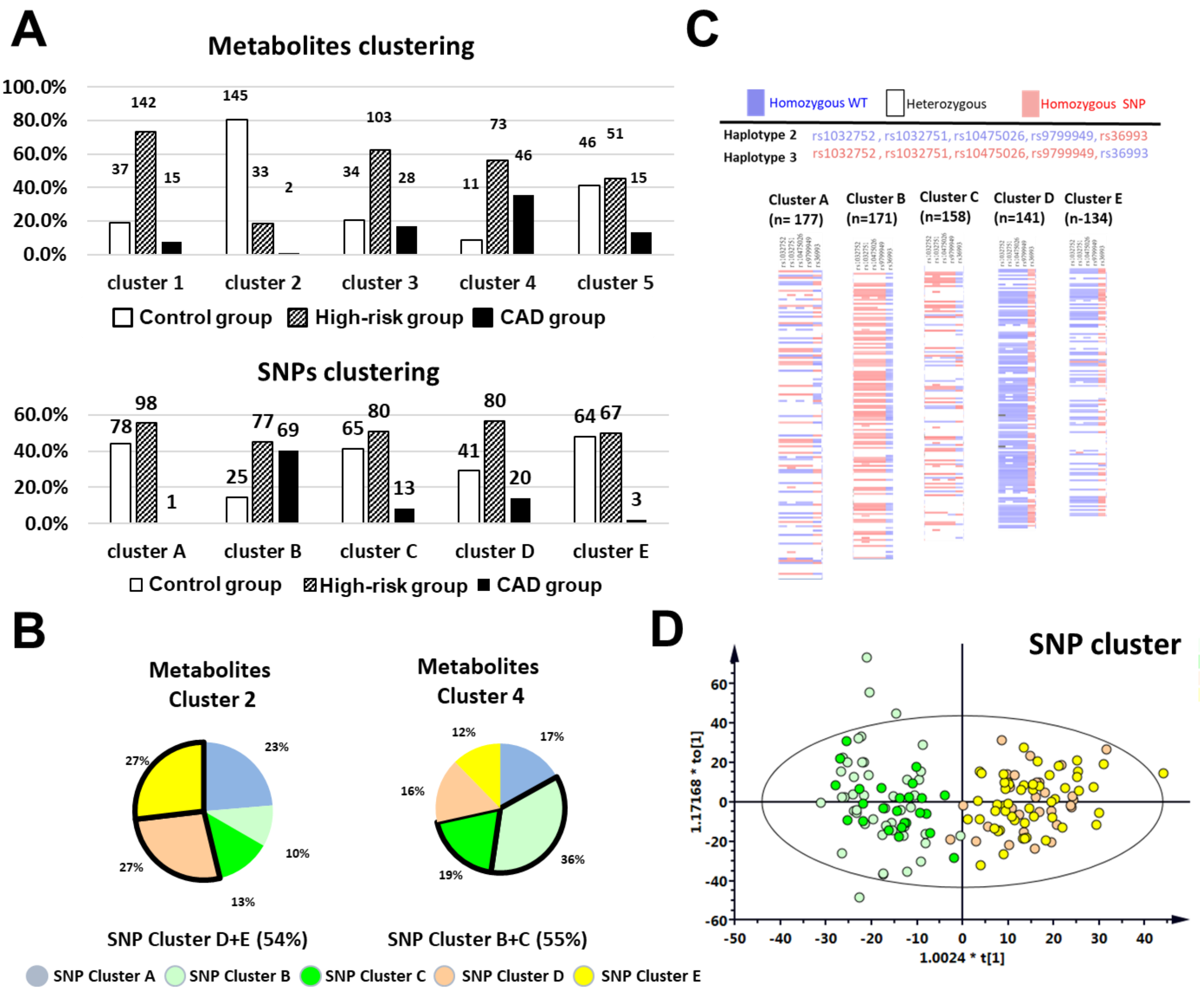

Figure S5. Combination of metabolomic and SNP clusters. **(A)** Distribution of three groups (Control, High-risk, CAD) in metabolites clustering (cluster 1 to 5) and SNP clustering (cluster A to D). In metabolites cluster 2, a higher proportion of subjects are the control group, whereas in metabolites cluster 4 most subjects are high-risk or CAD. **(B)** In metabolites cluster 2, most subjects belong to SNP cluster D and E (Chi-square test, $p = 2.5 \times 10^{-19}$) , while in metabolites cluster 4, most subjects belong to SNP cluster B and C (Chi-square test, $p = 1.13 \times 10^{-13}$). **(C)** Haplotype 2 was mainly observed in subjects from clusters D and E, while haplotype 3 was observed in clusters B and C. **(D)** Comparison of subjects from SNP clusters B to E using orthogonal partial least squares-discriminant analysis (OPLS-DA) plot representing that SNP clusters B and C are close together, while SNP clusters D and E are close together. The 143 variables and 151 observations were selected to build this OPLS-DA score plot by cross-validation rules. The sum of squares capture by this model (R2) was 0.814 and the cross-validated (Q2) was 0.755. **(E)** Metabolomics clusters: Cluster 4 shows the highest CAD risk, with a BMI of 28.1, predominantly male (0.65). Cluster 2 is the healthiest, with a BMI of 22.9, average age of 59.7, and a slight female dominance (0.37). **(F)** SNP clusters: Cluster B, at highest CAD risk, has a BMI of 26.7, average age of 63.6, and is predominantly male (0.55). Cluster E, with the lowest risk, has a BMI of 25.2 and a more balanced gender ratio (0.42).

Figure S5 continued

E

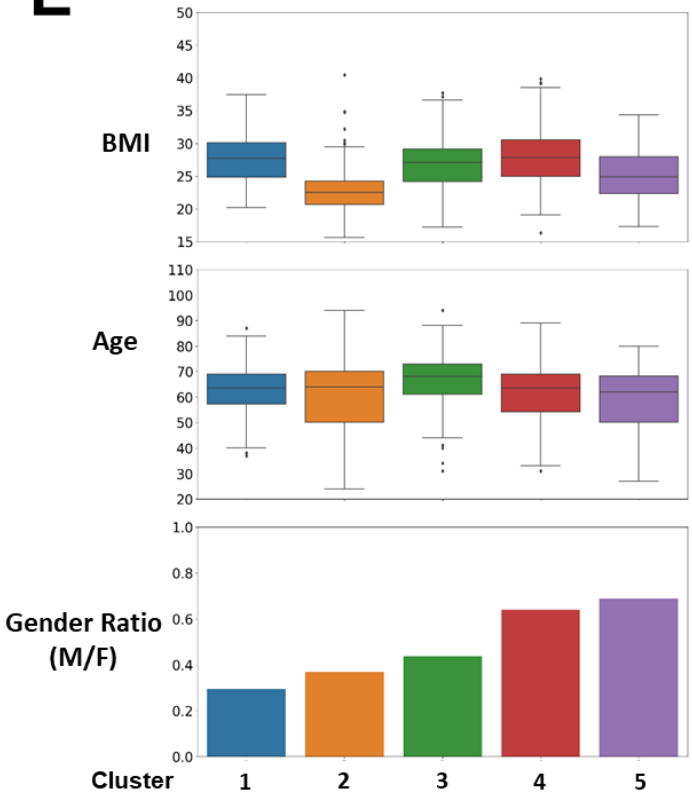

Comparison	BMI P-value	Age P-value	Sex P-value
Cluster 1 vs Cluster 2	0.00E+00	1.38E-01	1.65E-01
Cluster 1 vs Cluster 3	8.46E-02	7.63E-04	7.04E-03
Cluster 1 vs Cluster 4	4.57E-01	3.11E-01	1.70E-09
Cluster 1 vs Cluster 5	3.13E-07	1.86E-02	1.00E-10
Cluster 2 vs Cluster 3	0.00E+00	2.71E-05	2.26E-01
Cluster 2 vs Cluster 4	0.00E+00	6.60E-01	4.00E-06
Cluster 2 vs Cluster 5	7.88E-08	3.97E-01	1.86E-07
Cluster 3 vs Cluster 4	3.69E-02	1.45E-04	8.57E-04
Cluster 3 vs Cluster 5	6.10E-04	1.92E-06	6.56E-05
Cluster 4 vs Cluster 5	6.24E-07	2.05E-01	5.04E-01

P-values < 0.05 are highlighted in red

F

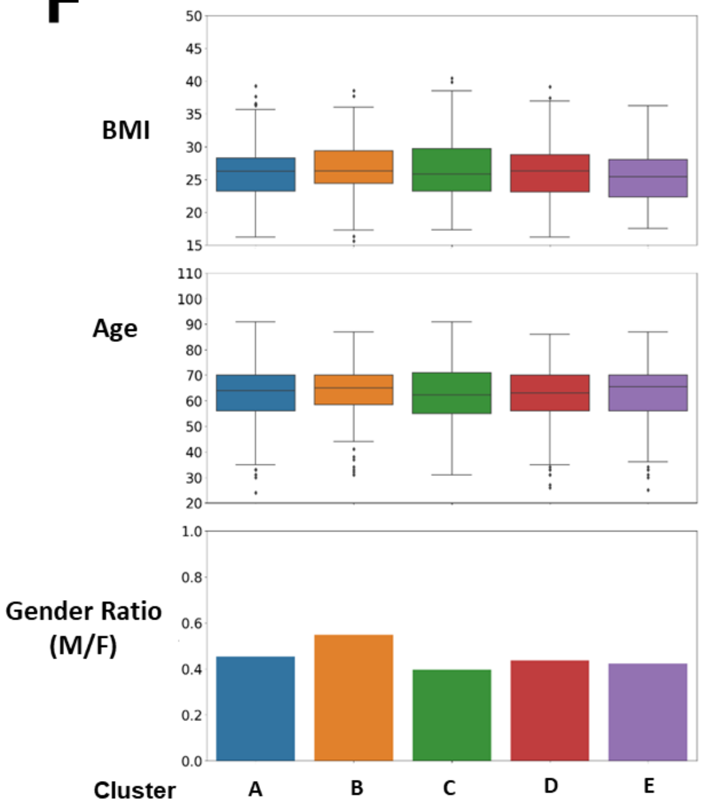

Comparison	BMI P-value	Age P-value	Sex P-value
Cluster A vs Cluster B	1.06E-01	4.73E-01	2.46E-02
Cluster A vs Cluster C	4.21E-01	6.16E-01	5.72E-01
Cluster A vs Cluster D	6.10E-01	5.78E-01	9.29E-01
Cluster A vs Cluster E	1.71E-01	7.11E-01	1.59E-01
Cluster B vs Cluster C	3.77E-01	2.37E-01	3.14E-03
Cluster B vs Cluster D	4.31E-01	1.91E-01	2.40E-02
Cluster B vs Cluster E	3.89E-03	8.03E-01	1.53E-04
Cluster C vs Cluster D	8.64E-01	8.74E-01	7.92E-01
Cluster C vs Cluster E	4.80E-02	4.93E-01	1.75E-01
Cluster D vs Cluster E	6.97E-02	3.56E-01	1.71E-01

P-values < 0.05 are highlighted in red

Figure S6

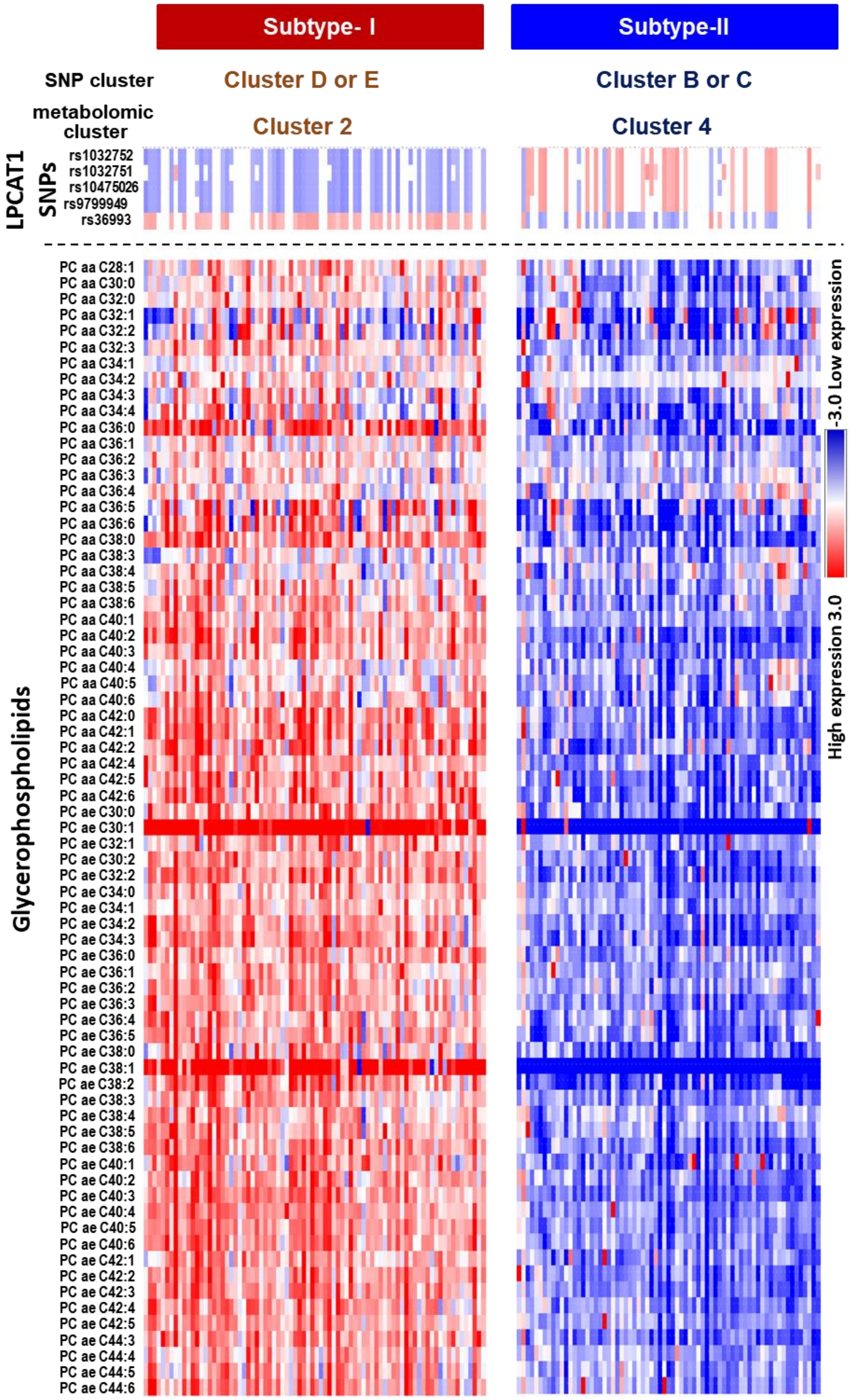

Figure S6. Bi-allelic SNPs of LPCAT1 were presented with homozygous allele 2 (SNP, red), homozygous allele 1 (WT, blue), and heterozygous (white) reading. The pattern of glycerophospholipids detected with targeted metabolomic abundance (mean cantered and divided by the range of each variable) in these two clusters of subjects aligned with SNPs were presented.

Figure S7

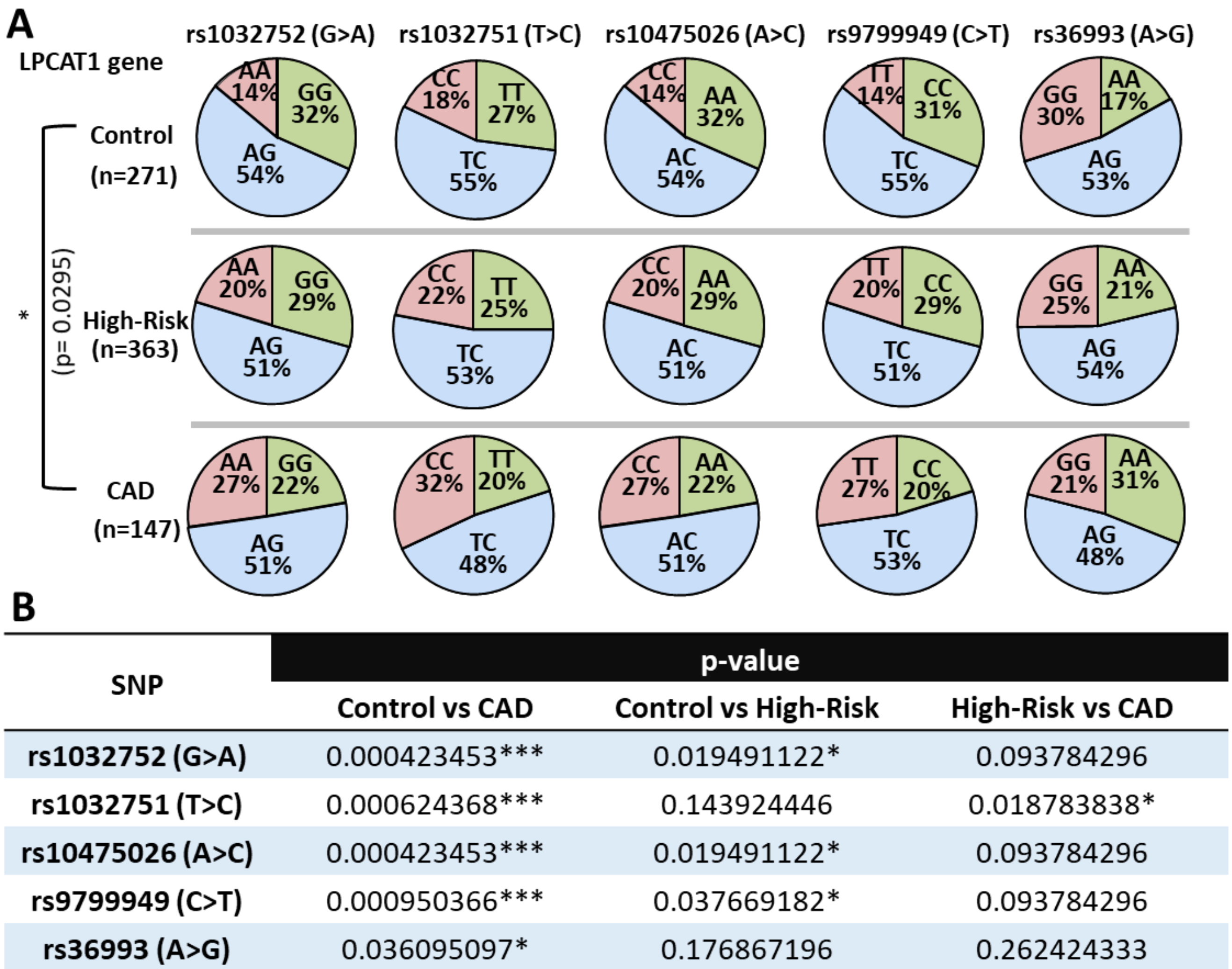

B

SNP	p-value		
	Control vs CAD	Control vs High-Risk	High-Risk vs CAD
rs1032752 (G>A)	0.000423453***	0.019491122*	0.093784296
rs1032751 (T>C)	0.000624368***	0.143924446	0.018783838*
rs10475026 (A>C)	0.000423453***	0.019491122*	0.093784296
rs9799949 (C>T)	0.000950366***	0.037669182*	0.093784296
rs36993 (A>G)	0.036095097*	0.176867196	0.262424333

Figure S7. (**A) Genotype frequencies of five LPCAT1 SNPs (rs1032752, rs1032751, rs10475026, rs9799949, rs36993) across Control, High Risk, and CAD groups.** ANOVA reveals a significant difference among the three groups ($p = 0.0365$). Proportion test shows significant differences between the Control and CAD groups ($p = 0.0295$). The figure suggests potential linkage disequilibrium between these SNPs, indicating a possible haplotype linkage that may contribute to the genetic predisposition to CAD and warrants further investigation. **(B) The p values of proportion test for SNPs in the LPCAT1 gene.** Significant differences are observed between the Control and CAD groups for rs1032752, rs1032751, rs10475026, and rs9799949 ($p < 0.001$). The SNP rs36993 also shows a significant difference ($p < 0.05$). Data are presented as the mean ± SD and analyzed by one-way ANOVA with Dunnett's correction. *** ($p \leq 0.001$; highly significant); ** ($0.001 < p \leq 0.01$; very significant); * ($0.01 < p \leq 0.05$; significant); $p > 0.05$ (not significant)

Figure S8

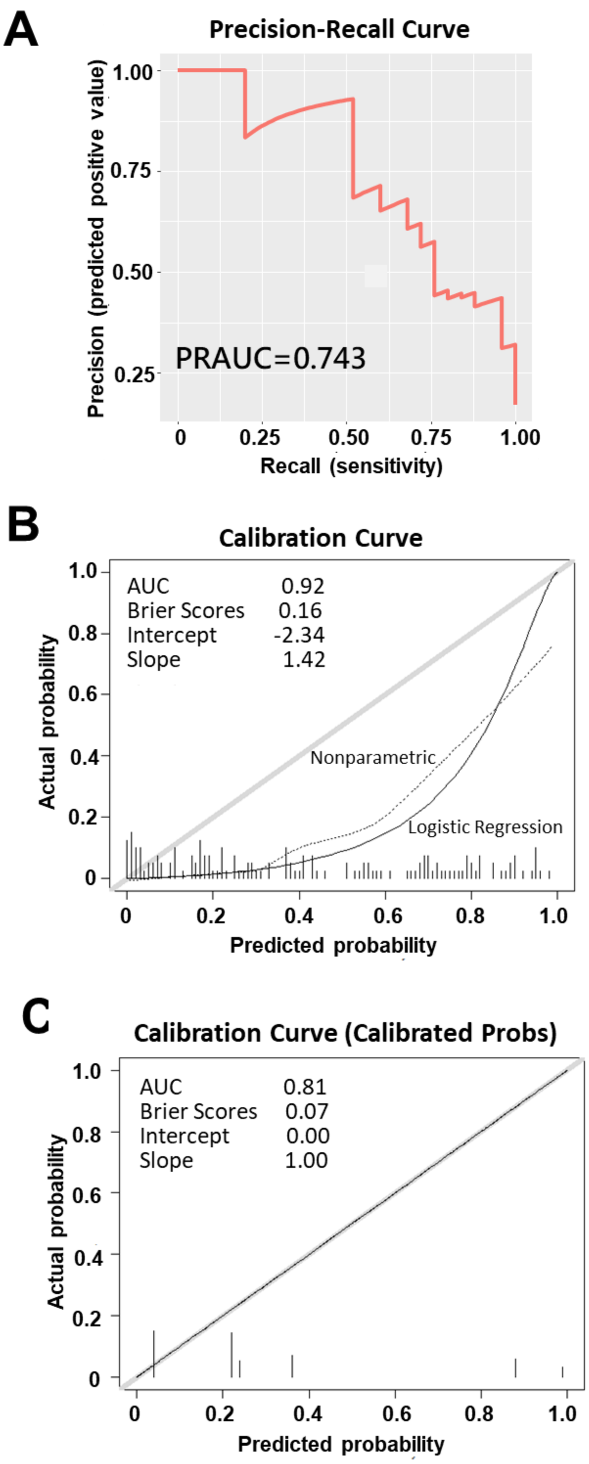

Figure S8. The evaluation matrices for machine learning model. (A) PRAUC (Precision Recall Area Under Curve), higher is better. **(B)** Calibration curve (before calibration) is used to evaluate the fitness between model predict probability and observed probability. **(C)** Calibration curve (after calibration). The intercept is zero and the slope is 1, seems fit well, but has lower AUC 0.81

Figure S9 STROBE Statement—Checklist of items that should be included in reports of ***cross-sectional studies***

	Item No	Recommendation	Page No
**Title and abstract**	1	(*a*) Indicate the study's design with a commonly used term in the title or the abstract	1
		(*b*) Provide in the abstract an informative and balanced summary of what was done and what was found	3
**Introduction**			
Background/rationale	2	Explain the scientific background and rationale for the investigation being reported	5
Objectives	3	State specific objectives, including any prespecified hypotheses	6
**Methods**			
Study design	4	Present key elements of study design early in the paper	6-7
Setting	5	Describe the setting, locations, and relevant dates, including periods of recruitment, exposure, follow-up, and data collection	6-7
Participants	6	(*a*) Give the eligibility criteria, and the sources and methods of selection of participants	6-7
Variables	7	Clearly define all outcomes, exposures, predictors, potential confounders, and effect modifiers. Give diagnostic criteria, if applicable	6-7
Data sources/ measurement	8*	For each variable of interest, give sources of data and details of methods of assessment (measurement). Describe comparability of assessment methods if there is more than one group	*6-10*
Bias	9	Describe any efforts to address potential sources of bias	6-7
Study size	10	Explain how the study size was arrived at	6-7
Quantitative variables	11	Explain how quantitative variables were handled in the analyses. If applicable, describe which groupings were chosen and why	6-7
Statistical methods	12	(*a*) Describe all statistical methods, including those used to control for confounding	10-11
		(*b*) Describe any methods used to examine subgroups and interactions	10-11
		(*c*) Explain how missing data were addressed	NA
		(*d*) If applicable, describe analytical methods taking account of sampling strategy	NA
		(*e*) Describe any sensitivity analyses	10-11
**Results**			
Participants	13*	(a) Report numbers of individuals at each stage of study—eg numbers potentially eligible, examined for eligibility, confirmed eligible, included in the study, completing follow-up, and analysed	11
		(b) Give reasons for non-participation at each stage	NA
		(c) Consider use of a flow diagram	6
Descriptive data	14*	(a) Give characteristics of study participants (eg demographic, clinical, social) and information on exposures and potential confounders	11
		(b) Indicate number of participants with missing data for each variable of interest	NA
Outcome data	15*	Report numbers of outcome events or summary measures	17
Main results	16	(*a*) Give unadjusted estimates and, if applicable, confounder-adjusted estimates and their precision (eg, 95% confidence interval). Make clear which confounders were adjusted for and why they were included	17
		(*b*) Report category boundaries when continuous variables were categorized	17
		(*c*) If relevant, consider translating estimates of relative risk into absolute risk for a meaningful time period	NA
Other analyses	17	Report other analyses done—eg analyses of subgroups and interactions, and sensitivity analyses	NA
**Discussion**			
Key results	18	Summarise key results with reference to study objectives	18
Limitations	19	Discuss limitations of the study, taking into account sources of potential bias or imprecision. Discuss both direction and magnitude of any potential bias	23
Interpretation	20	Give a cautious overall interpretation of results considering objectives, limitations, multiplicity of analyses, results from similar studies, and other relevant evidence	25
Generalisability	21	Discuss the generalisability (external validity) of the study results	25
**Other information**			
Funding	22	Give the source of funding and the role of the funders for the present study and, if applicable, for the original study on which the present article is based	25

*Give information separately for exposed and unexposed groups.

Note: An Explanation and Elaboration article discusses each checklist item and gives methodological background and published examples of transparent reporting. The STROBE checklist is best used in conjunction with this article (freely available on the Web sites of PLoS Medicine at http://www.plosmedicine.org/, Annals of Internal Medicine at http://www.annals.org/, and Epidemiology at http://www.epidem.com/). Information on the STROBE Initiative is available at www.strobe-statement.org.

Supplemental Tables

Table S1. The 143 targeted metabolites was quantified and processed for plasma metabolites cluster.

Amino acids	Biogenic amines	Acylcarnitines		Phospholipid		Sphingolipids
Ala	ADMA	C0	lysoPC a C16:0	PC aa C28:1	PC ae C30:0	SM (OH) C14:1
Arg	Creatinine	C2	lysoPC a C16:1	PC aa C30:0	PC ae C30:1	SM (OH) C16:1
Asn	Kynurenine	C3	lysoPC a C17:0	PC aa C32:0	PC ae C30:2	SM (OH) C22:1
Asp	Putrescine	C4	lysoPC a C18:0	PC aa C32:1	PC ae C32:1	SM (OH) C22:2
Cit	Sarcosine	C5	lysoPC a C18:1	PC aa C32:2	PC ae C32:2	SM (OH) C24:1
Gln	SDMA	C5:1-DC	lysoPC a C18:2	PC aa C32:3	PC ae C34:0	SM C16:0
Glu	Serotonin	C7-DC	lysoPC a C20:3	PC aa C34:1	PC ae C34:1	SM C16:1
Gly	t4-OH-Pro	C8	lysoPC a C20:4	PC aa C34:2	PC ae C34:2	SM C18:0
His	Taurine	C10	lysoPC a C24:0	PC aa C34:3	PC ae C34:3	SM C18:1
Ile		C12	lysoPC a C26:0	PC aa C34:4	PC ae C36:0	SM C20:2
Leu		C14:1	lysoPC a C26:1	PC aa C36:0	PC ae C36:1	SM C24:0
Lys		C16	lysoPC a C28:0	PC aa C36:1	PC ae C36:2	SM C24:1
Met		C18	lysoPC a C28:1	PC aa C36:2	PC ae C36:3	SM C26:0
Orn		C18:1		PC aa C36:3	PC ae C36:4	SM C26:1
Phe		C18:2		PC aa C36:4	PC ae C36:5	
Pro				PC aa C36:5	PC ae C38:0	
Ser				PC aa C36:6	PC ae C38:1	
Thr				PC aa C38:0	PC ae C38:2	
Trp				PC aa C38:3	PC ae C38:3	
Tyr				PC aa C38:4	PC ae C38:4	
Val				PC aa C38:5	PC ae C38:5	
				PC aa C38:6	PC ae C38:6	
				PC aa C40:1	PC ae C40:1	
				PC aa C40:2	PC ae C40:2	
				PC aa C40:3	PC ae C40:3	
				PC aa C40:4	PC ae C40:4	
				PC aa C40:5	PC ae C40:5	
				PC aa C40:6	PC ae C40:6	
				PC aa C42:0	PC ae C42:1	
				PC aa C42:1	PC ae C42:2	
				PC aa C42:2	PC ae C42:3	
				PC aa C42:4	PC ae C42:4	
				PC aa C42:5	PC ae C42:5	
				PC aa C42:6	PC ae C44:3	
					PC ae C44:4	
					PC ae C44:5	
					PC ae C44:6	

Table S2. Performance of 75 hyperparameter optimization combinations for XGBoost model

eta	max_depth	min_child_weight	Sensitivity Mean (sd)	Specificity Mean (sd)	PPV Mean (sd)	NPV Mean (sd)	Accuracy Mean (sd)	AUC Mean (sd)
0.1	6	4	0.836 (0.072)	0.813 (0.042)	0.495 (0.058)	0.959 (0.017)	0.817 (0.033)	0.908 (0.025)
0.1	12	4	0.836 (0.072)	0.813 (0.042)	0.495 (0.058)	0.959 (0.017)	0.817 (0.033)	0.908 (0.025)
0.1	30	4	0.836 (0.072)	0.813 (0.042)	0.495 (0.058)	0.959 (0.017)	0.817 (0.033)	0.908 (0.025)
0.1	12	2	0.839 (0.064)	0.812 (0.045)	0.495 (0.062)	0.960 (0.015)	0.816 (0.037)	0.907 (0.027)
0.1	30	2	0.839 (0.064)	0.812 (0.045)	0.495 (0.062)	0.960 (0.015)	0.816 (0.037)	0.907 (0.027)
0.1	12	1	0.834 (0.072)	0.808 (0.043)	0.487 (0.059)	0.958 (0.017)	0.813 (0.036)	0.906 (0.027)
0.1	30	1	0.834 (0.072)	0.808 (0.043)	0.487 (0.059)	0.958 (0.017)	0.813 (0.036)	0.906 (0.027)
0.1	6	2	0.838 (0.064)	0.811 (0.045)	0.494 (0.062)	0.959 (0.015)	0.816 (0.037)	0.906 (0.027)
0.1	6	1	0.832 (0.069)	0.809 (0.043)	0.488 (0.060)	0.958 (0.017)	0.813 (0.036)	0.906 (0.026)
0.3	6	1	0.833 (0.075)	0.810 (0.043)	0.490 (0.058)	0.958 (0.018)	0.814 (0.035)	0.904 (0.027)
0.1	6	8	0.809 (0.080)	0.810 (0.042)	0.482 (0.055)	0.953 (0.018)	0.810 (0.033)	0.904 (0.025)
0.1	12	8	0.809 (0.080)	0.810 (0.042)	0.482 (0.055)	0.953 (0.018)	0.810 (0.033)	0.904 (0.025)
0.1	30	8	0.809 (0.080)	0.810 (0.042)	0.482 (0.055)	0.953 (0.018)	0.810 (0.033)	0.904 (0.025)
0.3	12	2	0.835 (0.069)	0.811 (0.045)	0.493 (0.062)	0.959 (0.016)	0.815 (0.036)	0.903 (0.028)
0.3	30	2	0.835 (0.069)	0.811 (0.045)	0.493 (0.062)	0.959 (0.016)	0.815 (0.036)	0.903 (0.028)
0.3	12	1	0.831 (0.071)	0.810 (0.045)	0.491 (0.064)	0.958 (0.017)	0.814 (0.038)	0.903 (0.027)
0.3	30	1	0.831 (0.071)	0.810 (0.045)	0.491 (0.064)	0.958 (0.017)	0.814 (0.038)	0.903 (0.027)
0.3	6	2	0.834 (0.067)	0.811 (0.045)	0.492 (0.062)	0.958 (0.016)	0.815 (0.036)	0.903 (0.027)
0.5	6	1	0.830 (0.068)	0.809 (0.043)	0.488 (0.054)	0.957 (0.016)	0.813 (0.034)	0.902 (0.027)
0.5	12	1	0.832 (0.073)	0.808 (0.043)	0.486 (0.055)	0.958 (0.017)	0.812 (0.035)	0.902 (0.027)
0.5	30	1	0.832 (0.073)	0.808 (0.043)	0.486 (0.055)	0.958 (0.017)	0.812 (0.035)	0.902 (0.027)
0.5	6	2	0.832 (0.065)	0.809 (0.044)	0.489 (0.056)	0.958 (0.015)	0.813 (0.034)	0.902 (0.027)
0.3	6	4	0.822 (0.069)	0.810 (0.044)	0.488 (0.058)	0.956 (0.016)	0.813 (0.034)	0.902 (0.027)
0.3	12	4	0.822 (0.069)	0.810 (0.044)	0.488 (0.058)	0.956 (0.016)	0.813 (0.034)	0.902 (0.027)
0.3	30	4	0.822 (0.069)	0.810 (0.044)	0.488 (0.058)	0.956 (0.016)	0.813 (0.034)	0.902 (0.027)
0.1	6	10	0.806 (0.081)	0.812 (0.043)	0.484 (0.052)	0.952 (0.019)	0.811 (0.033)	0.902 (0.026)
0.1	12	10	0.806 (0.081)	0.812 (0.043)	0.484 (0.052)	0.952 (0.019)	0.811 (0.033)	0.902 (0.026)
0.1	30	10	0.806 (0.081)	0.812 (0.043)	0.484 (0.052)	0.952 (0.019)	0.811 (0.033)	0.902 (0.026)
0.5	12	2	0.832 (0.065)	0.809 (0.044)	0.489 (0.057)	0.958 (0.015)	0.813 (0.034)	0.901 (0.027)
0.5	30	2	0.832 (0.065)	0.809 (0.044)	0.489 (0.057)	0.958 (0.015)	0.813 (0.034)	0.901 (0.027)
0.7	12	1	0.832 (0.073)	0.808 (0.042)	0.486 (0.058)	0.958 (0.018)	0.812 (0.035)	0.900 (0.028)
0.7	30	1	0.832 (0.073)	0.808 (0.042)	0.486 (0.058)	0.958 (0.018)	0.812 (0.035)	0.900 (0.028)
0.5	6	4	0.823 (0.074)	0.809 (0.044)	0.485 (0.057)	0.956 (0.017)	0.811 (0.035)	0.900 (0.026)
0.5	12	4	0.823 (0.074)	0.809 (0.044)	0.485 (0.057)	0.956 (0.017)	0.811 (0.035)	0.900 (0.026)
0.5	30	4	0.823 (0.074)	0.809 (0.044)	0.485 (0.057)	0.956 (0.017)	0.811 (0.035)	0.900 (0.026)
0.3	6	8	0.805 (0.080)	0.811 (0.043)	0.482 (0.053)	0.952 (0.018)	0.810 (0.032)	0.900 (0.026)
0.3	12	8	0.805 (0.080)	0.811 (0.043)	0.482 (0.053)	0.952 (0.018)	0.810 (0.032)	0.900 (0.026)
0.3	30	8	0.805 (0.080)	0.811 (0.043)	0.482 (0.053)	0.952 (0.018)	0.810 (0.032)	0.900 (0.026)
0.3	6	10	0.810 (0.079)	0.810 (0.045)	0.483 (0.053)	0.953 (0.018)	0.810 (0.034)	0.900 (0.025)
0.3	12	10	0.810 (0.079)	0.810 (0.045)	0.483 (0.053)	0.953 (0.018)	0.810 (0.034)	0.900 (0.025)
0.3	30	10	0.810 (0.079)	0.810 (0.045)	0.483 (0.053)	0.953 (0.018)	0.810 (0.034)	0.900 (0.025)
0.7	6	1	0.829 (0.074)	0.807 (0.043)	0.485 (0.056)	0.957 (0.018)	0.811 (0.035)	0.899 (0.029)
0.7	6	2	0.833 (0.068)	0.809 (0.045)	0.490 (0.060)	0.958 (0.016)	0.814 (0.038)	0.899 (0.028)
0.7	12	2	0.831 (0.068)	0.809 (0.045)	0.488 (0.060)	0.958 (0.016)	0.813 (0.037)	0.899 (0.028)
0.7	30	2	0.831 (0.068)	0.809 (0.045)	0.488 (0.060)	0.958 (0.016)	0.813 (0.037)	0.899 (0.028)
0.9	12	1	0.826 (0.070)	0.808 (0.042)	0.485 (0.055)	0.956 (0.017)	0.812 (0.034)	0.898 (0.028)
0.9	30	1	0.826 (0.070)	0.808 (0.042)	0.485 (0.055)	0.956 (0.017)	0.812 (0.034)	0.898 (0.028)
0.9	6	1	0.826 (0.073)	0.809 (0.042)	0.487 (0.057)	0.957 (0.017)	0.812 (0.034)	0.897 (0.027)
0.5	6	10	0.807 (0.080)	0.811 (0.043)	0.483 (0.050)	0.952 (0.018)	0.810 (0.032)	0.897 (0.024)
0.5	12	10	0.807 (0.080)	0.811 (0.043)	0.483 (0.050)	0.952 (0.018)	0.810 (0.032)	0.897 (0.024)
0.5	30	10	0.807 (0.080)	0.811 (0.043)	0.483 (0.050)	0.952 (0.018)	0.810 (0.032)	0.897 (0.024)
0.7	6	4	0.821 (0.077)	0.808 (0.043)	0.483 (0.058)	0.955 (0.018)	0.810 (0.035)	0.895 (0.029)
0.7	12	4	0.821 (0.077)	0.808 (0.043)	0.483 (0.058)	0.955 (0.018)	0.810 (0.035)	0.895 (0.029)
0.7	30	4	0.821 (0.077)	0.808 (0.043)	0.483 (0.058)	0.955 (0.018)	0.810 (0.035)	0.895 (0.029)
0.9	6	2	0.816 (0.077)	0.808 (0.045)	0.482 (0.056)	0.954 (0.018)	0.810 (0.035)	0.895 (0.028)
0.9	12	2	0.816 (0.077)	0.808 (0.045)	0.482 (0.056)	0.954 (0.018)	0.810 (0.035)	0.895 (0.028)
0.9	30	2	0.816 (0.077)	0.808 (0.045)	0.482 (0.056)	0.954 (0.018)	0.810 (0.035)	0.895 (0.028)
0.7	6	10	0.808 (0.086)	0.807 (0.045)	0.478 (0.052)	0.952 (0.020)	0.807 (0.033)	0.895 (0.026)
0.7	12	10	0.808 (0.086)	0.807 (0.045)	0.478 (0.052)	0.952 (0.020)	0.807 (0.033)	0.895 (0.026)
0.7	30	10	0.808 (0.086)	0.807 (0.045)	0.478 (0.052)	0.952 (0.020)	0.807 (0.033)	0.895 (0.026)
0.5	6	8	0.807 (0.071)	0.806 (0.043)	0.476 (0.052)	0.952 (0.016)	0.806 (0.033)	0.895 (0.025)
0.5	12	8	0.807 (0.071)	0.806 (0.043)	0.476 (0.052)	0.952 (0.016)	0.806 (0.033)	0.895 (0.025)
0.5	30	8	0.807 (0.071)	0.806 (0.043)	0.476 (0.052)	0.952 (0.016)	0.806 (0.033)	0.895 (0.025)
0.9	6	4	0.818 (0.071)	0.807 (0.039)	0.480 (0.053)	0.955 (0.017)	0.809 (0.031)	0.891 (0.029)
0.9	12	4	0.818 (0.071)	0.807 (0.039)	0.480 (0.053)	0.955 (0.017)	0.809 (0.031)	0.891 (0.029)
0.9	30	4	0.818 (0.071)	0.807 (0.039)	0.480 (0.053)	0.955 (0.017)	0.809 (0.031)	0.891 (0.029)
0.9	6	10	0.799 (0.080)	0.800 (0.044)	0.466 (0.050)	0.950 (0.018)	0.800 (0.033)	0.891 (0.027)
0.9	12	10	0.799 (0.080)	0.800 (0.044)	0.466 (0.050)	0.950 (0.018)	0.800 (0.033)	0.891 (0.027)
0.9	30	10	0.799 (0.080)	0.800 (0.044)	0.466 (0.050)	0.950 (0.018)	0.800 (0.033)	0.891 (0.027)
0.7	6	8	0.799 (0.077)	0.802 (0.044)	0.469 (0.055)	0.950 (0.018)	0.802 (0.035)	0.890 (0.025)
0.7	12	8	0.799 (0.077)	0.802 (0.044)	0.469 (0.055)	0.950 (0.018)	0.802 (0.035)	0.890 (0.025)
0.7	30	8	0.799 (0.077)	0.802 (0.044)	0.469 (0.055)	0.950 (0.018)	0.802 (0.035)	0.890 (0.025)
0.9	6	8	0.797 (0.079)	0.798 (0.044)	0.463 (0.050)	0.949 (0.018)	0.798 (0.034)	0.889 (0.026)
0.9	12	8	0.797 (0.079)	0.798 (0.044)	0.463 (0.050)	0.949 (0.018)	0.798 (0.034)	0.889 (0.026)
0.9	30	8	0.797 (0.079)	0.798 (0.044)	0.463 (0.050)	0.949 (0.018)	0.798 (0.034)	0.889 (0.026)

PPV, positive predictive value; NPV, negative predictive value; AUC, area under the receiver operating characteristic curve

Table S3. Performance comparison of Random forest, XGBoost and optimal XGBoost models

Method	Sensitivity Mean (SD)	Specificity Mean (SD)	PPV Mean (SD)	NPV Mean (SD)	Accuracy Mean (SD)	AUC Mean (SD)
**RF**	**0.856 (0.061)**	**0.806 (0.042)**	**0.491 (0.053)**	**0.964 (0.014)**	**0.815 (0.032)**	**0.917 (0.022)**
**XGBoost**	0.837 (0.071)	0.809 (0.044)	0.491 (0.062)	0.959 (0.017)	0.814 (0.036)	0.904 (0.026)
**XGBoost Optimal**	0.836 (0.071)	0.813 (0.042)	0.495 (0.058)	0.960 (0.017)	0.817 (0.033)	0.908 (0.025)

RF, random forest; PPV, positive predictive value; NPV, negative predictive value; AUC, area under curve; SD, standard deviation.

Table S4. 100 times Bootstrapped validation and 10-fold cross validation of prediction performance in Random Forest models

Method	Sensitivity Mean (SD)	Specificity Mean (SD)	PPV Mean (SD)	NPV Mean (SD)	Accuracy Mean (SD)	AUC Mean (SD)
**RF** Bootstrap	**0.856 (0.061)**	**0.806 (0.042)**	**0.491 (0.053)**	**0.964 (0.014)**	**0.815 (0.032)**	**0.917 (0.022)**
**RF** 10-folds cross-validation	**0.879 (0.063)**	**0.803 (0.051)**	**0.491 (0.064)**	**0.969 (0.016)**	**0.816 (0.042)**	**0.922 (0.026)**

RF, random forest; PPV, positive predictive value; NPV, negative predictive value; AUC, area under curve; SD, standard deviation.

Table S5. 10-fold cross validation performance with selected features in each fold

Fold	Sensitivity	Specificity	PPV	NPV	Accuracy	AUC	Selected Features
1	0.86	0.75	0.43	0.96	0.77	0.89	**Sarcosine, Aspartate, Creatinine,** PC_aa_C36_0, **Serotonin, PC_ae_C34_3**, Glucose
2	0.85	0.74	0.52	0.96	0.84	0.92	**Sarcosine, Aspartate, Proline, Creatinine, Serotonin,** PC_ae_C36_3, PC_aa_C32_2, PC_ae_C34_3
3	0.85	0.78	0.44	0.96	0.79	0.91	**Sarcosine, Creatinine, Aspartate, Serotonin,** PC_aa_C32_2, **PC_ae_C34_3,** PC_ae_C36_3, **PC_aa_C40_2**
4	0.85	0.78	0.44	0.96	0.79	0.87	**Sarcosine, Aspartate, Creatinine, Serotonin,** PC_ae_C36_3, PC_ae_C30_0, **PC_ae_C34_3**, PC_aa_C36_6
5	1.00	0.78	0.48	1.00	0.82	0.97	**Sarcosine, Aspartate, Creatinine, PC_ae_C34_3, Serotonin**, PC_aa_C32_2, PC_ae_C36_3, **Proline** Glucose
6	0.77	0.85	0.53	0.95	0.84	0.91	**Sarcosine, Aspartate, PC_ae_C34_3, Creatinine**, **Serotonin**, PC_ae_C36_3, **Proline,** PC_ae_C32_2 Glucose
7	0.77	0.86	0.53	0.95	0.84	0.93	**Sarcosine, Creatinine, Aspartate, PC_ae_C34_3, Proline, Serotonin**, PC_aa_C32_2, PC_ae_C36_3
8	1.00	0.79	0.50	1.00	0.83	0.95	**Sarcosine, Aspartate, Creatinine, Proline, Serotonin, PC_ae_C34_3**, PC_aa_C32_2, PC_ae_C36_3, Glucose, PC_ae_C30_0
9	0.77	0.81	0.45	0.94	0.80	0.88	**Sarcosine, Aspartate, PC_ae_C34_3, Creatinine, Serotonin, Proline**, PC_ae_C36_3, PC_aa_C32_2
10	0.86	0.75	0.43	0.96	0.77	0.88	**Sarcosine, Creatinine, Aspartate, Serotonin,** PC_aa_C32_2, **PC_ae_C34_3**
**Mean**	**0.86**	**0.80**	**0.48**	**0.96**	**0.81**	**0.91**	Not Appropriate

Features with Bold are the same with bootstrapped method

Table S6. Basic characteristics of participants in each metabolomic cluster.

Cluster	1	2	3	4	5	
Number	194	180	165	130	112	*p*
Sex (male, %)	57 (29.4)	66 (36.7)	72 (43.6)	84 (64.6)	77 (68.8)	<0.0001
Age (years, mean)	63.2 ± 10	59.7 ± 15.1	66.7 ± 11.2	61.2 ± 11.9	58.5 ± 13.7	<0.0001
Group						<0.0001
Control (%)	36 (18.6)	145 (80.6)	33 (20)	11 (8.5)	46 (41.1)	
High-risk (%)	131 (67.5)	28 (15.6)	102 (61.8)	67 (51.5)	35 (31.3)	
CAD (%)	27 (13.9)	7 (3.9)	30 (18.2)	52 (40)	31 (27.7)	
DM (%)	61 (31.4)	22 (12.2)	57 (34.5)	74 (56.9)	35 (31.5)	<0.0001
CKD (%)	52 (26.8)	12 (6.7)	52 (31.5)	49 (37.7)	25 (22.5)	<0.0001
Height (cm)	157 ± 8.2	157.9 ± 8.1	158.1 ± 8.8	162.3 ± 9.1	164.3 ± 9	<0.0001
Weight (Kg)	68.2 ± 11.6	57.3 ± 11	67.3 ± 12.5	74.4 ± 15.3	68 ± 12.8	<0.0001
BMI	27.6 ± 3.6	22.9 ± 3.4	26.9 ± 4	28.1 ± 4.6	25.2 ± 3.8	<0.0001
Waist (cm)	88.7 ± 9.1	76.6 ± 9.8	88.1 ± 10.1	93.1 ± 11.9	84.7 ± 11.9	<0.0001
Systolic BP (mmHg)	144.2 ± 84.9	119.8 ± 15	135.7 ± 16.2	134 ± 19.3	126.8 ± 19.1	<0.0001
Diastolic BP (mmHg)	80.1 ± 10.3	71.3 ± 8.7	78.3 ± 10.4	78 ± 12.3	74.4 ± 11.7	<0.0001
Heart rate (BMP)	76.3 ± 12.6	73.9 ± 11.7	76.7 ± 11.9	78.3 ± 14.1	71.2 ± 12.9	<0.0001
Fasting glucose (mg/dL)	112.1 ± 36.8	95 ± 16.5	111.9 ± 38.6	132.7 ± 56.9	109.4 ± 47.5	<0.0001
Glycohemoglobin (%)	6.3 ± 1.2	5.7 ± 0.7	6.8 ± 7.9	6.8 ± 1.5	6 ± 0.9	0.038
Total cholesterol (mg/dL)	198.8 ± 39.8	203 ± 31.1	185.5 ± 33.9	155.2 ± 29.5	170.8 ± 37.5	<0.0001
HDL (mg/dL)	52.5 ± 12.3	67.9 ± 16.6	54 ± 15.1	42.4 ± 10.9	51.6 ± 13.5	<0.0001
LDL (mg/dL)	134.1 ± 40.1	127.1 ± 31.2	118 ± 31.9	97.8 ± 30.1	109.5 ± 32.6	<0.0001
Triglyceride (mg/dL)	155.6 ± 104.1	91.4 ± 60.1	155 ± 94.8	146.4 ± 77.3	121.4 ± 62.6	<0.0001
BUN (mg/dL)	16.6 ± 10	15.5 ± 7.4	17.7 ± 7.5	18.5 ± 21.3	16.6 ± 6.8	0.191
Creatinine (mg/dL)	0.9 ± 0.3	0.8 ± 0.4	1.3 ± 4.7	1.1 ± 1.4	1 ± 0.9	0.218
Albumin (g/dL)	4.6 ± 0.3	4.6 ± 0.3	4.5 ± 0.4	4.4 ± 0.9	4.5 ± 0.3	0.320
ALT (U/L)	28.8 ± 17	22.6 ± 15.5	26.4 ± 14.4	33.2 ± 21.8	28.6 ± 24.9	<0.0001
Total bilirubin (mg/dL)	0.6 ± 0.2	0.7 ± 0.3	0.6 ± 0.3	0.6 ± 0.5	0.7 ± 0.3	0.067
Alkaline phosphatase (U/L)	75.9 ± 18.7	65.9 ± 19.5	74.8 ± 20.2	71.2 ± 26.3	67 ± 20	<0.0001
RBC (10^6/μL)	4.7 ± 0.6	4.7 ± 0.6	4.7 ± 0.6	4.8 ± 0.7	4.7 ± 0.6	0.419
Hemoglobin (g/dL)	14.1 ± 1.3	13.6 ± 1.4	13.8 ± 1.8	14.1 ± 1.7	13.9 ± 1.6	0.011
WBC (10^3/μL)	6.6 ± 2.6	5.7 ± 1.7	6.4 ± 2	7.1 ± 2.1	6.4 ± 1.9	<0.0001
Platelets (10^3/μL)	280.5 ± 74.5	260.3 ± 56.5	249.5 ± 57.8	257.9 ± 72.9	246.6 ± 57.8	<0.0001
Adiponectin (μg/mL)	8.0 ± 7.2	8.1 ± 8.0	10.0 ± 7.7	5.9 ± 5.5	7.4 ± 7.4	0.021
Leptin (ng/mL)	14.0 ± 12.7	6.8 ± 6.6	11.2 ± 8.8	9.2 ± 9.4	8.1 ± 5.7	<0.0001
HS-CRP (mg/L)	2.6 ± 4.6	2.1 ± 5.7	3.1 ± 8.8	6.0 ± 22.9	4.3 ± 21.7	0.164
**Medications**						
Statin (%)	47 (24.2)	28 (15.6)	45 (27.3)	54 (41.5)	35 (31.3)	<0.0001
Fibrate (%)	5 (2.6)	0 (0)	3 (1.8)	0 (0)	6 (5.4)	0.006
CCB (%)	25 (12.9)	13 (7.2)	19 (11.5)	22 (16.9)	24 (21.4)	0.007
β-blocker (%)	29 (14.9)	19 (10.6)	17 (10.3)	24 (18.5)	24 (21.4)	0.032
ARB (%)	39 (20.1)	17 (9.4)	30 (18.2)	27 (20.8	25 (22.3)	0.021
Diuretics (%)	8 (4.1)	5 (2.8)	13 (7.9)	9 (6.9)	8 (7.1)	0.192

CAD, coronary artery disease; BP, Blood pressure; BMI, body mass index; DM, diabetes mellitus; CKD, chronic kidney disease; HDL, high-density lipoprotein; LDL, low-density lipoprotein; BUN, blood urea nitrogen; ALT, alanine aminotransferase; RBC, red blood cell; WBC, white blood cell; HS-CRP, high-sensitive C-Reactive protein; CCB, calcium channel blocker; ARB, angiotensin receptor blocker. Data are presented as the mean ± SD and analyzed by one-way ANOVA with Dunnett's correction.

Table S7. Top ten significant metabolites in differentiation of participants among control, high-risk, and CAD groups.

Rank	metabolites	Classes	p-value
1	PC ae C40:3	Glycerophospholipids	1.58E-38
2	PC ae C30:1	Glycerophospholipids	1.94E-27
3	PC ae C38:6	Glycerophospholipids	2.27E-25
4	PC ae C38:3	Glycerophospholipids	4.9E-25
5	PC ae C40:5	Glycerophospholipids	1.57E-23
6	PC ae C42:2	Glycerophospholipids	8.53E-21
7	PC ae C38:1	Glycerophospholipids	4.76E-18
8	SM (OH) C14:1	Sphingomyelins	3.37E-16
9	PC aa C40:3	Glycerophospholipids	1.6E-14
10	PC aa C42:6	Glycerophospholipids	4.26E-14

Table S8. Genotype frequencies of various SNPs in control, high-risk, and CAD groups.

Gene	SNP	Control (n=271)			High Risk (n=363)			CAD (n=147)		
**LPCAT1**	rs1032752 (G>A)	GG	AG	AA	GG	AG	AA	GG	AG	AA
		0.32	0.55	0.14	0.29	0.5	0.2	0.22	0.5	0.27
	rs1032751 (T>C)	TT	TC	CC	TT	TC	CC	TT	TC	CC
		0.27	0.55	0.18	0.25	0.53	0.22	0.2	0.48	0.32
	rs10475026 (A>C)	AA	AC	CC	AA	AC	CC	AA	AC	CC
		0.32	0.55	0.14	0.29	0.5	0.2	0.22	0.5	0.27
	rs9799949 (C>T)	CC	TC	TT	CC	TC	TT	CC	TC	TT
		0.31	0.55	0.14	0.29	0.51	0.2	0.2	0.52	0.27
	rs36993 (A>G)	AA	AG	GG	AA	AG	GG	AA	AG	GG
		0.17	0.53	0.3	0.21	0.53	0.25	0.31	0.48	0.21
**ATP13A2**	rs2311528 (G>A)	GG	AG	AA	GG	AG	AA	GG	AG	AA
		0.11	0.52	0.37	0.18	0.48	0.34	0.2	0.52	0.28
	Affx-5865445 (C>T)	CC	TC	TT	CC	TC	TT	CC	TC	TT
		0.12	0.54	0.35	0.19	0.49	0.32	0.22	0.52	0.26
	rs2076602 (A>T)	TT	AT	AA	TT	AT	AA	TT	AT	AA
		0.11	0.54	0.35	0.18	0.5	0.32	0.22	0.52	0.25
**SH3GL2**	rs1998246 (A>G)	GG	AG	AA	GG	AG	AA	GG	AG	AA
		0.24	0.48	0.28	0.25	0.45	0.3	0.12	0.46	0.41
	rs6475158 (A>G)	GG	AG	AA	GG	AG	AA	GG	AG	AA
		0.27	0.46	0.27	0.28	0.45	0.26	0.41	0.47	0.12
	rs12349497 (G>T)	GG	TG	TT	GG	TG	TT	GG	TG	TT
		0.28	0.46	0.26	0.31	0.45	0.23	0.45	0.44	0.12
	rs2209440 (T>G)	GG	TG	TT	GG	TG	TT	GG	TG	TT
		0.34	0.5	0.16	0.38	0.46	0.16	0.24	0.5	0.25
**VAMP8**	rs7579147 (G>A)	GG	AG	AA	GG	AG	AA	GG	AG	AA
		0.52	0.42	0.06	0.47	0.43	0.1	0.37	0.53	0.1
**TULP4**	rs9348183 (C>T)	CC	TC	TT	CC	TC	TT	CC	TC	TT
		0.24	0.45	0.3	0.32	0.48	0.2	0.36	0.46	0.18
	rs9348214 (A>G)	GG	AG	AA	GG	AG	AA	GG	AG	AA
		0.24	0.45	0.3	0.32	0.48	0.2	0.36	0.46	0.18

SNP, Single Nucleotide Polymorphisms; CAD, coronary artery disease.

Table S9. Top 10 important SNPs of K-means clustering

			Current study (n=781)				Taiwan biobank (n=1,517)
Rank	SNPs	Genes	p-value (chi-squared test)	p-value (SAIGE)	Frequency (%)	Ref	Frequency (%)
1	rs9799949	LPCAT1	5.97E-03	2.49E-03	CC:28.2 CT:52.3 TT:19.5	C	CC:29.7 CT:50.1 TT:20.2
2	rs1032752	LPCAT1	1.28E-02	4.21E-03	GG:28.7 AG:51.7 AA:19.6	G	GG:19.2 AG:50.0 AA:30.7
3	rs1998246	SH3GL2	2.18E-02	2.44E-03	AA:22.0 AG:46.4 GG:31.2	A	AA:20.7 AG:51.0 GG:28.3
4	rs36993	LPCAT1	2.70E-02	6.33E-03	AA:21.5 AG:52.4 GG:26.1	A	AA:21.7 AG:50.3 GG:28.0
5	rs6475158	SH3GL2	1.37E-02	3.20E-04	AA:30.2 AG:45.6 GG:24.1	A	AA:27.4 AG:50.9 GG:21.7
6	rs7579147	VAMP8	2.71E-02	1.54E-02	GG:46.6 AG:44.8 AA:8.6	G	GG:46.7 AG:42.3 AA:11.0
7	rs9348214	TULP4	3.30E-02	6.91E-02	AA:29.8 AG:46.7 GG:23.3	A	AA:29.4 AG:49.0 GG:21.6
8	rs2076602	ATP13A2	3.15E-03	2.24E-02	AA:16.1 AT:52.0 TT:31.9	A	AA:18.0 AT:50.5 TT:31.5
9	Affx-5865445	ATP13A2	2.94E-02	N/A	CC:17.0 CT:51.1 TT:31.8	C	no record
10	rs2311528	ATP13A2	1.21E-02	7.20E-02	GG:15.7 AG:50.4 AA:33.8	G	GG:16.3 AG:50.2 AA:33.4

Table S10. Basic characteristics of subjects in different clusters according to SNP analysis

Cluster	A	B	C	D	E	p
n	177	171	158	141	134	
Sex (male, %)	81 (45.8)	94 (55.0)	63 (39.9)	62 (44.0)	56 (41.8)	0.059
Age (years, mean)	62.2±12.7	63.6±11.5	61.7±13.1	60.8±13.2	61.9±13.2	0.403
**Group** (n)						<0.0001
Control (%)	78 (44.1)	24 (14)	64 (40.5)	41 (29.1)	64 (47.8)	
High-risk (%)	93 (52.5)	66 (38.6)	75 (47.5)	73 (51.8)	56 (41.8)	
CAD (%)	6 (3.4)	81 (47.4)	19 (12)	27 (19.1)	14 (10.4)	
DM (%)	48 (27.1)	87 (51.2)	43 (27.2)	39 (27.7)	32 (23.9)	<0.0001
CKD (%)	43 (24.3)	65 (38.2)	38 (24.1)	19 (13.5)	25 (18.7)	<0.0001
Height (cm)	158.7±8.6	160.8±9.3	158.8±8.3	159.7±9.4	158.6±9.3	0.112
Weight (Kg)	65.3±12.7	69.3±13.5	66.7±14	67±14.9	63.7±12.9	0.006
BMI	25.9±4.3	26.7±4.1	26.3±4.6	26.1±4.6	25.2±3.9	0.035
Waist (cm)	85±10.6	89.3±12	85.9±11.8	85.2±12.4	83.5±11.5	0.001
Systolic BP (mmHg)	130.1±17.6	133.8±20.1	130.2±18.8	140.9±100.6	128.7±16.6	0.159
Diastolic BP (mmHg)	77.1±11.1	76.5±10.9	76±11.8	78±11.1	74.9±10.1	0.195
Heart rate (BMP)	76.1±13.3	75.9±13.6	74.6±11.9	75.1±12.6	75.2±11.9	0.836
Fasting glucose (mg/dL)	107.1±34.1	123.3±50.5	110.3±46.5	109.8±41.5	103.6±23.6	<0.0001
Glycohemoglobin (%)	6.1±1.1	7.2±8	6.2±1.2	6.1±0.9	6±0.8	0.029
Total cholesterol (mg/dL)	192.4±35.1	172.5±42.7	184.8±36.7	186.7±35.8	193.7±39.2	<0.0001
HDL (mg/dL)	55.2±15	49.1±15.7	54.9±14.5	56.1±17.8	58.8±17	<0.0001
LDL (mg/dL)	126.1±34.2	109.6±37.5	118.5±32.4	120.2±35.9	123.9±38.3	<0.0001
Triglyceride (mg/dL)	126.4±70.3	149±77.9	134.1±112.4	133.2±84.9	127.4±86.5	0.14
BUN (mg/dL)	15.9±5.4	19.9±20.9	16±5.9	16.8±9.6	15.5±5.3	0.004
Creatinine (mg/dL)	0.9±0.3	1.5±4.8	0.9±0.3	0.9±0.5	0.8±0.3	0.023
Albumin (g/dL)	4.6±0.2	4.5±1.2	4.5±1.4	4.5±0.3	4.6±0.3	0.398
ALT (U/L)	25.8±13.4	31.9±26.3	26.8±17.1	26±13	26.9±19.4	0.017
Total bilirubin (mg/dL)	0.6±0.3	0.7±0.5	0.6±0.3	0.6±0.3	0.7±0.3	0.861
Alkaline phosphatase (U/L)	72.4±19.6	71.4±23.8	70.3±19.7	71.9±21.9	70.2±20.8	0.602
RBC (10^6/μL)	4.7±0.5	4.7±0.7	4.7±0.6	4.7±0.6	4.7±0.5	0.766
Hemoglobin (g/dL)	14.1±1.4	13.8±1.7	13.9±1.5	13.7±1.5	13.8±1.5	0.098
WBC (10^3/μL)	6.3±2.1	6.7±2.2	6.5±1.9	6.2±1.9	6.1±2.6	0.132
Platelets (10^3/μL)	261±74.1	252.5±66.7	262.2±63.1	268±62.8	261.3±57.7	0.361
Adiponectin (μg/mL)	8.2 ± 8.6	7.8 ± 6.9	7.5 ± 5.8	9.2 ± 9.2	7.1 ± 5.5	0.481
Leptin (ng/mL)	10.9 ± 11.1	9.8 ± 8.1	12.2 ± 11.0	7.1 ± 6.4	10.3 ± 9.7	0.014
HS-CRP (mg/L)	1.9 ± 2.5	6.3 ± 26.6	2.4 ± 5.9	4.0 ± 8.4	2.4 ± 5.1	0.037
**Medications**						
Statin (%)	34 (19.2)	78 (45.6)	31 (19.6)	38 (27.0)	28 (20.9)	<0.0001
Fibrate (%)	4 (2.3)	3 (1.8)	2 (1.3)	3 (2.1)	2 (1.5)	0.961
CCB (%)	19 (10.7)	24 (14.0)	17 (10.8)	24 (17.0)	19 (14.2)	0.445
β-blocker (%)	19 (10.7)	41 (24.0)	16 (10.1)	21 (14.9)	16 (11.9)	0.002
ARB (%)	28 (15.8)	44 (25.7)	23 (14.6)	19 (13.5)	24 (17.9)	0.03
Diuretics (%)	8 (4.5)	16 (9.4)	7 (4.4)	9 (6.4)	3 (2.2)	0.075

CAD, coronary artery disease; BP, Blood pressure; BMI, body mass index; DM, diabetes mellitus; CKD, chronic kidney disease; HDL, high-density lipoprotein; LDL, low-density lipoprotein; BUN, blood urea nitrogen; ALT, alanine aminotransferase; RBC, red blood cell; WBC, white blood cell; HS-CRP, high-sensitive C-Reactive protein; CCB, calcium channel blocker; ARB, angiotensin receptor blocker. Data are presented as the mean ± SD and analyzed by one-way ANOVA with Dunnett's correction.

Table S11. Comparison of genotype frequencies for CAD-associated SNPs reported previously from CARDIoGRAMplusC4D Consortium across the control, high-risk, and CAD groups.

Gene	SNP	Risk allele frequency (risk allele)	Reference	In this study (Axiom Genome-Wide TWB 2.0 Array)									Risk match*
				Control			High-risk			CAD			
ZC3HC1	rs11556924 (C>T)	0.62 (C)	Schunkert et al., 2011[66]	0.89 (CC)	0.10 (CT)	0.00 (TT)	0.87 (CC)	0.12 (CT)	0.01 (TT)	0.93 (CC)	0.07 (CT)	0.00 (TT)	No
ABO	rs579459 (C>T)	0.21 (C)		0.03 (CC)	0.24 (CT)	0.72 (TT)	0.02 (CC)	0.30 (CT)	0.67 (TT)	0.02 (CC)	0.38 (CT)	0.60 (TT)	No
CYP17A1, CNNM2, NT5C2	rs12413409 (G>A)	0.89 (G)		0.50 (GG)	0.41 (GA)	0.08 (AA)	0.53 (GG)	0.41 (GA)	0.06 (AA)	0.56 (GG)	0.38 (GA)	0.06 (AA)	No
ZNF259, APOA5-A4-C3-A1	rs964184 (G>C)	0.13 (G)		0.04 (GG)	0.27 (GC)	0.68 (CC)	0.04 (GG)	0.36 (GC)	0.60 (CC)	0.03 (GG)	0.30 (GC)	0.67 (CC)	No
COL4A1, COL4A2	rs4773144 (A>G)	0.44 (G)		0.39 (AA)	0.46 (AG)	0.15 (GG)	0.34 (AA)	0.52 (AG)	0.14 (GG)	0.35 (AA)	0.54 (AG)	0.10 (GG)	No
HHIPL1	rs2895811 (T>C)	0.43 (C)		0.59 (TT)	0.35 (TC)	0.06 (CC)	0.59 (TT)	0.37 (TC)	0.04 (CC)	0.59 (TT)	0.35 (TC)	0.07 (CC)	No
ADAMTS7	rs3825807 (A>G)	0.57 (A)		0.70 (AA)	0.26 (AG)	0.04 (GG)	0.70 (AA)	0.27 (AG)	0.02 (GG)	0.78 (AA)	0.21 (AG)	0.01 (GG)	No
SMG6, SRR	rs216172 (G>C)	0.37 (C)		0.49 (GG)	0.44 (GC)	0.07 (CC)	0.50 (GG)	0.43 (GC)	0.07 (CC)	0.56 (GG)	0.37 (GC)	0.06 (CC)	No
RASD1, SMCR3, PEMT	rs12936587 (G>A)	0.56 (G)		0.79 (GG)	0.20 (GT)	0.01 (TT)	0.82 (GG)	0.17 (GT)	0.02 (TT)	0.82 (GG)	0.16 (GT)	0.03 (TT)	No
UBE2Z, GIP, ATP5G1, SNF8	rs46522 (C>T)	0.53 (T)		0.11 (CC)	0.47 (CT)	0.41 (TT)	0.10 (CC)	0.45 (CT)	0.44 (TT)	0.13 (CC)	0.41 (CT)	0.46 (TT)	No
SORT1	rs599839 (G>A)	0.78 (A)		0.01 (GG)	0.15 (AG)	0.84 (AA)	0.00 (GG)	0.16 (AG)	0.83 (AA)	0.00 (GG)	0.16 (AG)	0.84 (AA)	No
PCSK9	rs11206510 (T>C)	0.82 (T)		0.90 (TT)	0.09 (TC)	0.01 (CC)	0.89 (TT)	0.11 (TC)	0.00 (CC)	0.90 (TT)	0.10 (TC)	0.00 (CC)	No
MIA3	rs17465637 (A>C)	0.74 (C)		0.17 (AA)	0.48 (AC)	0.36 (CC)	0.12 (AA)	0.52 (AC)	0.37 (CC)	0.13 (AA)	0.46 (AC)	0.41 (CC)	No
LPA	rs3798220 (T>C)	0.02 (C)		0.87 (TT)	0.13 (TC)	0.00 (CC)	0.83 (TT)	0.17 (TC)	0.01 (CC)	0.81 (TT)	0.18 (TC)	0.01 (CC)	No
CDKN2A/B, ANRIL	rs4977574 (A>G)	0.46 (G)		0.29 (AA)	0.50 (AG)	0.21 (GG)	0.28 (AA)	0.52 (AG)	0.19 (GG)	0.28 (AA)	0.53 (AG)	0.19 (GG)	No
CXCL12	rs1746048 (C>T)	0.87 (C)		0.49 (CC)	0.38 (TC)	0.13 (TT)	0.48 (CC)	0.44 (TC)	0.09 (TT)	0.52 (CC)	0.34 (TC)	0.14 (TT)	No
LDLR	rs1122608 (G>T)	0.77 (G)		0.79 (GG)	0.21 (TG)	0.01 (TT)	0.87 (GG)	0.12 (TG)	0.01 (TT)	0.82 (GG)	0.16 (TG)	0.01 (TT)	No
LIPA	rs1412444 (C>T)	0.42 (T)	Peden et al., 2011[67]	0.45 (CC)	0.42 (CT)	0.13 (TT)	0.49 (CC)	0.40 (CT)	0.10 (TT)	0.39 (CC)	0.53 (CT)	0.07 (TT)	No
ADAMTS7	rs4380028 (C>T)	0.65 (C)		0.27 (CC)	0.53 (TC)	0.20 (TT)	0.26 (CC)	0.52 (TC)	0.22 TT)	0.34 (CC)	0.50 (TC)	0.16 (TT)	No
7q22.3 (gene rich region)	rs10953541 (C>T)	0.80 (C)		0.71 (CC)	0.26 (CT)	0.03 (TT)	0.71 (CC)	0.26 (CT)	0.02 (TT)	0.74 (CC)	0.24 (CT)	0.01 (TT)	No
KIAA1462	rs2505083 (T>C)	0.38 (C)		0.59 (TT)	0.35 (TC)	0.06 (CC)	0.66 (TT)	0.30 (TC)	0.04 (CC)	0.62 (TT)	0.34 (TC)	0.03 (CC)	No
IL6R	rs4845625 (T>C)	0.47 (T)	Deloukas et al., 2013[68]	0.29 (TT)	0.50 (TC)	0.21 (CC)	0.21 (TT)	0.56 (TC)	0.23 (CC)	0.27 (TT)	0.48 (TC)	0.25 (CC)	No
APOB	rs515135 (T>C)	0.83 (C)		0.01 (TT)	0.17 (TC)	0.81 (CC)	0.01 (TT)	0.17 (TC)	0.83 (CC)	0.00 (TT)	0.18 (TC)	0.82 (CC)	No
VAMP5-VAMP8-GGCX	rs1561198 (C>T)	0.45 (T)		0.42 (CC)	0.47 (TC)	0.11 (TT)	0.38 (CC)	0.48 (TC)	0.13 (TT)	0.29 (CC)	0.58 (TC)	0.14 (TT)	No
SLC22A4-SLC22A5	rs273909 (A>G)	0.14 (G)		0.94 (AA)	0.06 (AG)	0.00 (GG)	0.93 (AA)	0.07 (AG)	0.01 (GG)	0.93 (AA)	0.07 (AG)	0.00 (GG)	No
KCNK5	rs10947789 (T>C)	0.76 (T)		0.66 (TT)	0.31 (TC)	0.03 (CC)	0.67 (TT)	0.31 (TC)	0.03 (CC)	0.69 (TT)	0.28 (TC)	0.03 (CC)	No
LPL	rs264 (G>A)	0.86 (G)		0.63 (GG)	0.32 (AG)	0.05 (AA)	0.58 (GG)	0.37 (AG)	0.04 (AA)	0.63 (GG)	0.32 (AG)	0.05 (AA)	No
FLT1	rs9319428 (G>A)	0.32 (A)		0.24 (GG)	0.54 (AG)	0.22 (AA)	0.29 (GG)	0.48 (AG)	0.22 (AA)	0.31 (GG)	0.46 (AG)	0.22 (AA)	No
REST-NOA1	rs17087335 (G>T)	0.21 (T)	Nikpay et al., 2015[4]	0.37 (GG)	0.42 (GT)	0.21 (TT)	0.37 (GG)	0.43 (GT)	0.19 (TT)	0.35 (GG)	0.48 (GT)	0.17 (TT)	No
PMAIP1-MC4R	rs663129 (G>A)	0.26 (A)		0.69 (GG)	0.27 (GA)	0.03 (AA)	0.67 (GG)	0.26 (GA)	0.05 (AA)	0.68 (GG)	0.31 (GA)	0.01 (AA)	No
KSR2	rs11830157 (T>G)	0.36 (G)		0.56 (TT)	0.35 (TG)	0.08 (GG)	0.50 (TT)	0.40 (TG)	0.10 (GG)	0.52 (TT)	0.41 (TG)	0.07 (GG)	No
ZNF507-LOC400684	rs12976411 (A>T)	0.09 (T)		0.31 (AA)	0.55 (AT)	0.14 (TT)	0.35 (AA)	0.48 (AT)	0.17 (TT)	0.40 (AA)	0.48 (AT)	0.12 (TT)	No
KCNJ13-GIGYF2	rs1801251 (G>A)	0.35 (A)	Webb et al., 2017[69]	0.44 (GG)	0.46 (GA)	0.11 (AA)	0.50 (GG)	0.37 (GA)	0.12 (AA)	0.53 (GG)	0.41 (GA)	0.06 (AA)	No
LRP1	rs11172113 (T>C)	0.41 (C)		0.57 (TT)	0.39 (TC)	0.04 (CC)	0.55 (TT)	0.40 (TC)	0.06 (CC)	0.49 (TT)	0.45 (TC)	0.06 (CC)	No
SCARB1	rs11057830 (G>A)	0.15 (A)		0.81 (GG)	0.18 (GA)	0.01 (AA)	0.80 (GG)	0.19 (GA)	0.01 (AA)	0.73 (GG)	0.24 (GA)	0.02 (AA)	No
CETP	rs1800775 (C>A)	0.51 (C)		0.23 (CC)	0.52 (CA)	0.25 (AA)	0.27 (CC)	0.47 (CA)	0.26 (AA)	0.16 (CC)	0.59 (CA)	0.24 (AA)	No

***Proportion test compares the frequency of risk allele between the control and CAD groups. p-value < 0.05 indicates "Yes" for statistical significance, otherwise "No".**

Table S11 (continued).

Gene	SNP	Risk allele frequency (risk allele)	Reference	In this study (Axiom Genome-Wide TWB 2.0 Array)									Risk match*
				Control			High risk			CAD			
PPAP2B	rs56170783 (A>C)	0.97 (A)		0.95 (AA)	0.05 (AC)	0.00 (CC)	0.94 (AA)	0.06 (AC)	0.00 (CC)	0.96 (AA)	0.03 (AC)	0.01 (CC)	No
CELSR2	rs12740374 (G>T)	0.93 (T)		0.01 (GG)	0.12 (GT)	0.87 (TT)	0.00 (GG)	0.13 (GT)	0.87 (TT)	0.00 (GG)	0.13 (GT)	0.87 (TT)	No
FLJ12334	rs16986953 (G>A)	0.69 (A)		0.06 (GG)	0.46 (GA)	0.47 (AA)	0.11 (GG)	0.46 (GA)	0.43 (AA)	0.08 (GG)	0.37 (GA)	0.54 (AA)	No
APOB	rs515135 (T>C)	0.09 (T)		0.01 (TT)	0.17 (TC)	0.81 (CC)	0.01 (TT)	0.17 (TC)	0.83 (CC)	0.00 (TT)	0.18 (TC)	0.82 (CC)	No
PARTICL	rs10176176 (A>T)	0.62 (A)		0.42 (AA)	0.48 (AT)	0.11 (TT)	0.39 (AA)	0.47 (AT)	0.14 (TT)	0.29 (AA)	0.58 (AT)	0.14 (TT)	No
EDNRA	rs6841581 (G>A)	0.80 (A)		0.03 (GG)	0.31 (GA)	0.66 (AA)	0.03 (GG)	0.33 (GA)	0.64 (AA)	0.05 (GG)	0.34 (GA)	0.61 (AA)	No
GUCY1A3	rs3796587 (C>G)	0.82 (C)		0.64 (CC)	0.32 (CG)	0.03 (GG)	0.71 (CC)	0.27 (CG)	0.02 (GG)	0.65 (CC)	0.31 (CG)	0.04 (GG)	No
ITGA1	rs4074793 (A>G)	0.94 (A)		0.88 (AA)	0.12 (AG)	0.00 (GG)	0.87 (AA)	0.12 (AG)	0.00 (GG)	0.88 (AA)	0.12 (AG)	0.00 (GG)	No
MAP3K1	rs3936510 (G>T)	0.90 (T)		0.01 (GG)	0.18 (GT)	0.81 (TT)	0.01 (GG)	0.18 (GT)	0.80 (TT)	0.01 (GG)	0.18 (GT)	0.82 (TT)	No
HMGCR	rs12916 (T>C)	0.47 (T)		0.22 (TT)	0.52 (TC)	0.26 (CC)	0.21 (TT)	0.52 (TC)	0.27 (CC)	0.21 (TT)	0.51 (TC)	0.28 (CC)	No
PHACTR1	rs9349379 (A>G)	0.31 (A)		0.08 (AA)	0.48 (AG)	0.43 (GG)	0.10 (AA)	0.44 (AG)	0.46 (GG)	0.05 (AA)	0.44 (AG)	0.51 (GG)	No
HDGFL1	rs6909752 (G>A)	0.83 (A)		0.03 (GG)	0.29 (GA)	0.68 (AA)	0.02 (GG)	0.26 (GA)	0.72 (AA)	0.02 (GG)	0.34 (GA)	0.64 (AA)	No
KCNK5	rs733701 (C>T)	0.87 (T)		0.02 (CC)	0.20 (CT)	0.79 (TT)	0.01 (CC)	0.23 (CT)	0.76 (TT)	0.02 (CC)	0.27 (CT)	0.71 (TT)	No
VEGFA	rs6905288 (G>A)	0.28 (A)		0.52 (GG)	0.41 (GA)	0.07 (AA)	0.53 (GG)	0.37 (GA)	0.10 (AA)	0.51 (GG)	0.41 (GA)	0.07 (AA)	No
TARID	rs6919211 (C>G)	0.76 (C)		0.60 (CC)	0.34 (CG)	0.06 (GG)	0.57 (CC)	0.36 (CG)	0.06 (GG)	0.59 (CC)	0.37 (CG)	0.04 (GG)	No
PLEKHG1	rs17080093 (C>T)	0.88 (T)		0.01 (CC)	0.21 (CT)	0.78 (TT)	0.02 (CC)	0.20 (CT)	0.78 (TT)	0.01 (CC)	0.18 (CT)	0.80 (TT)	No
LPA	rs73596816 (G>A)	0.95 (A)		0.00 (GG)	0.08 (GA)	0.91 (AA)	0.00 (GG)	0.08 (GA)	0.92 (AA)	0.00 (GG)	0.12 (GA)	0.88 (AA)	No
ZC3HC1	rs11556924 (C>T)	0.94 (T)		0.00 (CC)	0.10 (CT)	0.89 (TT)	0.01 (CC)	0.12 (CT)	0.87 (TT)	0.00 (CC)	0.07 (CT)	0.93 (TT)	No
CDKN2B-AS1	rs2891168 (A>G)	0.54 (A)	Aragam et al., 2022[5]	0.29 (AA)	0.49 (AG)	0.21 (GG)	0.28 (AA)	0.52 (AG)	0.20 (GG)	0.27 (AA)	0.53 (AG)	0.19 (GG)	No
ABO	rs651007 (T>C)	0.18 (T)		0.03 (TT)	0.24 (TC)	0.72 (CC)	0.02 (TT)	0.30 (TC)	0.67 (CC)	0.02 (TT)	0.38 (TC)	0.60 (CC)	No
TRIM5	rs11601507 (C>A)	0.89 (A)		0.01 (CC)	0.19 (CA)	0.80 (AA)	0.02 (CC)	0.20 (CA)	0.79 (AA)	0.01 (CC)	0.19 (CA)	0.80 (AA)	No
TMEM41B	rs4537761 (T>C)	0.88 (T)		0.76 (TT)	0.23 (TC)	0.00 (CC)	0.75 (TT)	0.23 (TC)	0.02 (CC)	0.85 (TT)	0.14 (TC)	0.01 (CC)	No
ZNF259	rs964184 (G>C)	0.20 (C)		0.68 (GG)	0.27 (GC)	0.04 (CC)	0.60 (GG)	0.36 (GC)	0.04 (CC)	0.67 (GG)	0.30 (GC)	0.03 (CC)	No
ATP2B1	rs2681472 (A>G)	0.67 (A)		0.40 (AA)	0.51 (AG)	0.09 (GG)	0.48 (AA)	0.42 (AG)	0.10 (GG)	0.43 (AA)	0.44 (AG)	0.12 (GG)	No
HNF1A	rs2244608 (A>G)	0.59 (A)		0.35 (AA)	0.46 (AG)	0.19 (GG)	0.38 (AA)	0.48 (AG)	0.14 (GG)	0.31 (AA)	0.48 (AG)	0.21 (GG)	No
DNAH10	rs7133378 (G>A)	0.88 (A)		0.02 (GG)	0.21 (GA)	0.76 (AA)	0.01 (GG)	0.21 (GA)	0.79 (AA)	0.00 (GG)	0.23 (GA)	0.76 (AA)	No
COL4A2	rs9515203 (T>C)	0.90 (T)		0.84 (TT)	0.14 (TC)	0.01 (CC)	0.78 (TT)	0.21 (TC)	0.01 (CC)	0.83 (TT)	0.16 (TC)	0.01 (CC)	No
MCF2L-AS1	rs4907571 (T>C)	0.77 (T)		0.57 (TT)	0.38 (TC)	0.05 (CC)	0.61 (TT)	0.35 (TC)	0.04 (CC)	0.59 (TT)	0.39 (TC)	0.03 (CC)	No
LIPC	rs588136 (C>T)	0.34 (T)		0.42 (CC)	0.48 (CT)	0.10 (TT)	0.44 (CC)	0.45 (CT)	0.11 (TT)	0.39 (CC)	0.49 (CT)	0.12 (TT)	No
MORF4L1	rs7173743 (T>C)	0.49 (T)		0.25 (TT)	0.46 (TC)	0.29 (CC)	0.21 (TT)	0.52 (TC)	0.28 (CC)	0.29 (TT)	0.52 (TC)	0.19 (CC)	No
CFDP1	rs8046696 (T>G)	0.48 (T)		0.25 (TT)	0.49 (TG)	0.25 (GG)	0.22 (TT)	0.50 (TG)	0.28 (GG)	0.18 (TT)	0.54 (TG)	0.27 (GG)	No
SMG6	rs4790881 (C>A)	0.60 (A)		0.16 (CC)	0.48 (CA)	0.36 (AA)	0.18 (CC)	0.47 (CA)	0.36 (AA)	0.15 (CC)	0.46 (CA)	0.39 (AA)	No
ANKRD13B	rs11080107 (T>C)	0.69 (T)		0.50 (TT)	0.40 (TC)	0.10 (CC)	0.46 (TT)	0.44 (TC)	0.10 (CC)	0.48 (TT)	0.44 (TC)	0.07 (CC)	No
MC4R	rs476828 (T>C)	0.80 (T)		0.67 (TT)	0.29 (TC)	0.04 (CC)	0.63 (TT)	0.31 (TC)	0.06 (CC)	0.65 (TT)	0.31 (TC)	0.04 (CC)	No
MYO9B	rs7246865 (G>A)	0.64 (A)		0.11 (GG)	0.46 (GA)	0.42 (AA)	0.14 (GG)	0.47 (GA)	0.38 (AA)	0.16 (GG)	0.37 (GA)	0.47 (AA)	No
B9D2	rs1800469 (A>G)	0.56 (A)		0.32 (AA)	0.49 (AG)	0.18 (GG)	0.32 (AA)	0.47 (AG)	0.21 (GG)	0.27 (AA)	0.51 (AG)	0.21 (GG)	No
OSM	rs6006426 (G>A)	0.45 (A)		0.25 (GG)	0.54 (GA)	0.21 (AA)	0.33 (GG)	0.48 (GA)	0.20 (AA)	0.32 (GG)	0.49 (GA)	0.19 (AA)	No

*Proportion test compares the frequency of risk allele between the control and CAD groups. p-value < 0.05 indicates "Yes" for statistical significance, otherwise "No".

Table S12. Basic characteristics of subjects in subtype-I and subtype-II.

Subtype	Subtype- I	Subtype- II	
Metabolomic Cluster	2	4	
SNP Cluster	D and E	B and C	
n	80	71	*p*
Sex (male, %)	28 (35.0)	48 (67.6)	
Age (years, mean)	58.7 ± 16	62.1 ± 11.4	0.132
**Group** (n)			**<0.0001**
Control (%)	65 (81.3)	3 (4.2)	
High-risk (%)	13 (16.3)	29 (40.8)	
CAD (%)	2 (2.5)	39 (54.9)	
DM (n, %)	7 (8.8)	44 (62)	**<0.0001**
CKD (n, %)	4 (5)	34 (47.9)	**<0.0001**
Height (cm)	157.8 ± 8.8	163.2 ± 8.5	**<0.0001**
Weight (Kg)	56.4 ± 10.7	74.1 ± 15.6	**<0.0001**
BMI	22.48 ± 2.75	27.62 ± 4.61	**<0.0001**
Waist (cm)	75.8 ± 9.2	94.1 ± 12.5	**<0.0001**
Systolic BP (mmHg)	120.4 ± 14.3	133.1 ± 20.6	**<0.0001**
Diastolic BP (mmHg)	72.1 ± 8.9	77.5 ± 11.4	**0.001**
Heart rate (BMP)	75.4 ± 11.2	79.6 ± 15.3	0.055
Fasting glucose (mg/dL)	94.2 ± 14	137.8 ± 64.5	**<0.0001**
Glycohemoglobin (%)	5.7 ± 0.5	7.0 ± 1.6	**<0.0001**
Total cholesterol (mg/dL)	197.4 ± 29.5	151.6 ± 30.5	**<0.0001**
HDL (mg/dL)	68.9 ± 17.1	41.5 ± 10.1	**<0.0001**
LDL (mg/dL)	120.2 ± 31	91.9 ± 31.4	**<0.0001**
Triglyceride (mg/dL)	95 ± 75.2	152.3 ± 82.8	**<0.0001**
BUN (mg/dL)	15.2 ± 7.7	17.5 ± 10.4	0.127
Creatinine (mg/dL)	0.80 ± 0.48	1.14 ± 1.32	**0.034**
Albumin (g/dL)	4.6 ± 0.3	4.9 ± 0.6	0.314
ALT (U/L)	23.1 ± 10.7	33.6 ± 20.5	**<0.0001**
Total bilirubin (mg/dL)	0.7 ± 0.3	0.7 ± 0.7	0.932
Alkaline phosphatase (U/L)	68.5 ± 23.1	69.8 ± 29.8	0.764
RBC (10^6/μL)	4.7 ± 0.5	4.7 ± 0.7	0.725
Hemoglobin (g/dL)	13.5 ± 1.4	13.9 ± 1.8	0.074
WBC (10^3/μL)	5.6 ± 1.7	7.4 ± 2.2	**<0.0001**
Platelets (10^3/μL)	258.9 ± 48.8	255.2 ± 70.3	0.703
Adiponectin (μg/mL)	7.4 ± 6.3	5.9 ± 6.6	0.328
Leptin (ng/mL)	5.4 ± 3.6	8.2 ± 5.7	**0.012**
HS-CRP (mg/L)	3 ± 8.3	7.7 ± 30.2	0.252
**Medications**			
Statin (%)	12 (15.0)	32(45.1)	**<0.0001**
Fibrate (%)	0 (0)	0 (0)	
CCB (%)	7 (8.8)	8 (11.3)	0.606
β-blocker (%)	8 (10.0)	16 (22.5)	**0.035**
ARB (%)	7 (8.8)	17 (23.9)	**0.011**
Diuretics (%)	2 (2.5)	7 (9.9)	0.057

CAD, coronary artery disease; BP, Blood pressure; BMI, body mass index; DM, diabetes mellitus; CKD, chronic kidney disease; HDL, high-density lipoprotein; LDL, low-density lipoprotein; BUN, blood urea nitrogen; ALT, alanine aminotransferase; RBC, red blood cell; WBC, white blood cell; HS-CRP, high-sensitive C-Reactive protein; CCB, calcium channel blocker; ARB, angiotensin receptor blocker. Data are presented as the mean ± SD and analyzed by one-way ANOVA with Dunnett's correction.

Table S13. 100 times Bootstrapped validation of prediction performance in Random Forest models (restricted on high-risk group and CAD group)

Method	Sensitivity Mean (SD)	Specificity Mean (SD)	PPV Mean (SD)	NPV Mean (SD)	Accuracy Mean (SD)	AUC Mean (SD)
**RF** Bootstrap	**0.860 (0.06)**	**0.700 (0.06)**	**0.510 (0.053)**	**0.930 (0.03)**	**0.740 (0.04)**	**0.880 (0.03)**

Table S14. Balance test for different prediction models

Randomly selected with the ratio of CAD and non-CAD as 1:1						
**Method**	**Sensitivity** Mean (SD)	**Specificity** Mean (SD)	**PPV** Mean (SD)	**NPV** Mean (SD)	**Accuracy** Mean (SD)	**AUC** Mean (SD)
**rf**	**0.856 (0.061)**	**0.813 (0.076)**	**0.824 (0.059)**	**0.854 (0.054)**	**0.835 (0.044)**	**0.919 (0.034)**
svm	0.855 (0.069)	0.781 (0.084)	0.799 (0.064)	0.850 (0.062)	0.818 (0.049)	0.906 (0.036)
tree	0.805 (0.097)	0.775 (0.086)	0.784 (0.064)	0.809 (0.080)	0.790 (0.056)	0.831 (0.055)
XGBoost	0.837 (0.071)	0.814 (0.071)	0.820 (0.057)	0.839 (0.060)	0.826 (0.046)	0.906 (0.036)
**Randomly selected with the ratio of CAD and non-CAD as 1:1 and matched with age and gender**						
**Method**	**Sensitivity** Mean (SD)	**Specificity** Mean (SD)	**PPV** Mean (SD)	**NPV** Mean (SD)	**Accuracy** Mean (SD)	**AUC** Mean (SD)
**rf**	**0.856 (0.061)**	**0.692 (0.097)**	**0.739 (0.060)**	**0.833 (0.058)**	**0.774 (0.049)**	**0.872 (0.040)**
svm	0.855 (0.069)	0.669 (0.115)	0.726 (0.067)	0.827 (0.068)	0.762 (0.059)	0.849 (0.050)
tree	0.805 (0.097)	0.708 (0.096)	0.737 (0.061)	0.795 (0.083)	0.756 (0.054)	0.802 (0.058)
XGBoost	0.837 (0.071)	0.708 (0.098)	0.745 (0.064)	0.818 (0.065)	0.772 (0.053)	0.860 (0.042)

CAD: coronary artery disease; RF: random forest; SVM: support vector machines; PPV: positive predictive value; NPV: negative predictive value; AUC: area under curve; SD: standard deviation

Table S15. Prediction performance in matched different medications

Balance CAD and non-CAD	Method	Sensitivity Mean (SD)	Specificity Mean (SD)	PPV Mean (SD)	NPV Mean (SD)	Accuracy Mean (SD)	AUC Mean (SD)
Randomly	rf	**0.856 (0.061)**	**0.813 (0.076)**	**0.824 (0.059)**	**0.854 (0.054)**	**0.835 (0.044)**	**0.919 (0.034)**
Match Statin	rf	**0.856 (0.061)**	**0.715 (0.078)**	**0.753 (0.051)**	**0.836 (0.06)**	**0.785 (0.047)**	**0.872 (0.039)**
Match Age, Gender	rf	**0.856 (0.061)**	**0.692 (0.097)**	**0.739 (0.060)**	**0.833 (0.058)**	**0.774 (0.049)**	**0.872 (0.040)**
Matching **Statin**, Age, Gender	rf	0.856 (0.061)	0.633 (0.102)	0.703 (0.058)	0.818 (0.065)	**0.744 (0.055)**	**0.842 (0.045)**
Matching **Fibrate**, Age, Gender	rf	0.856 (0.061)	0.698 (0.092)	0.742 (0.058)	0.834 (0.058)	0.777 (0.048)	0.876 (0.038)
Matching **β-blocker** , Age, Gender	rf	0.856 (0.061)	0.682 (0.106)	0.734 (0.065)	0.830 (0.060)	0.769 (0.053)	0.870 (0.042)
Matching **CCB**, Age, Gender	rf	0.856 (0.061)	0.686 (0.109)	0.737 (0.065)	0.830 (0.063)	0.771 (0.057)	0.870 (0.044)
Matching **ARB**, Age, Gender	rf	0.856 (0.061)	0.675 (0.101)	0.729 (0.060)	0.828 (0.061)	0.766 (0.053)	0.865 (0.042)
Matching **Diuretics**, Age, Gender	rf	0.856 (0.061)	0.689 (0.100)	0.738 (0.062)	0.832 (0.058)	0.772 (0.050)	0.869 (0.041)

CAD: coronary artery disease; RF: random forest; SVM: support vector machines; PPV: positive predictive value; NPV: negative predictive value; AUC: area under curve; SD: standard deviation; CCB: calcium channel blocker; ARB: angiotensin receptor blocker.

Table S16. Prediction performance of adding interaction features

Balance CAD and non-CAD	Method	Sensitivity Mean (SD)	Specificity Mean (SD)	PPV Mean (SD)	NPV Mean (SD)	Accuracy Mean (SD)	AUC Mean (SD)
Randomly	rf	**0.865 (0.076)**	**0.849 (0.067)**	**0.853 (0.059)**	**0.868 (0.066)**	**0.857 (0.052)**	**0.934 (0.033)**
Match Statin	rf	0.865 (0.076)	**0.526 (0.096)**	0.647 (0.046)	0.804 (0.088)	**0.695 (0.054)**	**0.795 (0.051)**
Match Age, Gender	rf	0.865 (0.076)	0.758 (0.087)	0.784 (0.062)	0.855 (0.072)	0.811 (0.057)	0.892 (0.04)
Matching Statin, Age, Gender	rf	0.865 (0.076)	**0.565 (0.084)**	0.667 (0.043)	0.816 (0.082)	0.715 (0.050)	0.81 (0.044)

CAD: coronary artery disease; RF: random forest; SVM: support vector machines; PPV: positive predictive value; NPV: negative predictive value; AUC: area under curve; SD: standard deviation

Table S17. Comparison of current risk assessment tools for identifying high-risk populations for coronary artery disease.

Test Type	Sensitivity (%)	Specificity (%)	Accuracy (%)	PPV (%)	NPV (%)	Reference
**Demographic-based prediction**						
**Framingham risk scores** [70]			AUC=0.68			Wannamethee et al, 2005
**Diamond-Forrester model** [71]			AUC=0.679			Miller et al. 2023
**Omics-based prediction**						
**This study (multi-omics)**			**AUC=0.917**			
**RNA and protein biomarkers**						
**Copeptin (204.1 pg/ml)** [72]	70.0	75.0	AUC=0.769			Cao et al. 2021
**Pentraxin3 (20.4 ng/ml)** [72]	70.0	75.0	AUC=0.781			Cao et al. 2021
**miR-296 and VEGF-β (severe)** [73]	77.9	66.7	AUC=0.783			Xu et al. 2023
**lnc-GAS5** [74]			AUC=0.915			Jiang et al. 2022
**miR-21** [74]			AUC=0.852			Jiang et al. 2022
**Blood biochemistry-based prediction**						
**γ-glutamyl transferase (IU/l) (cut-off=29.0)** [75]	44.4	79.6	AUC=0.636			Cho et al. 2019
**Uric acid (mg/dl) (cut-off=5.5)** [75]	47.0	75.5	AUC=0.605			Cho et al. 2019
**Hs-cTn-based proteomic mode*** [76]	80	71	AUC=0.85-0.86	83	66	McCarthy et al. 2020
**Image-based prediction**						
**Static Electrocardiogram** [70]	20-50	80-90	60-70	30-50	80-90	Wannamethee et al, 2005
**Exercise Treadmill Test** [77]	39	91	73	Low-Medium	Medium	Gulati et al. 2021
**Stress Echocardiography** [78]	70-90	77-90				Fihn et al. 2012
**Myocardial Perfusion Imaging** [79]	37-83	39-79	AUC=0.739	64	99	Tiwari et al. 2021
**Single-Photon Emission Computed Tomography** [80]	60-72	78-86		65-76	74-82	Greenwood et al. 2012
**Coronary Artery Calcium Scoring (>87; 50% stenosis)** [81]	62	76	64 (AUC=0.75)	91	33	Chen et al. 2023
**Coronary Artery Calcium Scoring (>290.8; 70% stenosis)** [81]	38	85	57 (AUC=0.66)	80	47	Chen et al. 2023
**Computed Tomography Coronary Angiography (>70% stenosis)** [82]	94	82	86 (AUC=0.92)	69	97	Nurmohamed et al. 2024
**Cardiovascular Magnetic Resonance** [80]	81.8-90.1	79.5-86.7		72.1-81.6	87.1-93.0	Greenwood et al. 2012
**Coronary Angiography** [83]	95-99	95-99	97	>95	>95	Lawton et al. 2021

*: including male sex, age, previous percutaneous coronary intervention, high-sensitivity cardiac troponin (hs-cTn), adiponectin, and kidney injury molecule-1; PPV, positive predictive value; NPV, negative predictive value; lnc-GAS5, Long-strand non-coding RNA Growth Arrest Specific 5; miR, microRNA

References cited only in the Supplementary Material.

66. Schunkert H, König IR, Kathiresan S, Reilly MP, Assimes TL, Holm H, et al. Large-scale association analysis identifies 13 new susceptibility loci for coronary artery disease. Nat Genet. 2011;43:333-8.
67. Coronary Artery Disease (C4D) Genetics Consortium: Peden JF, Hopewell JC, Saleheen D, Chambers JC, Hager J, Soranzo N, et al. A genome-wide association study in Europeans and South Asians identifies five new loci for coronary artery disease. Nat Genet. 2011;43:339-44.
68. CARDIoGRAMplusC4D Consortium; Deloukas P, Kanoni S, Willenborg C, Farrall M, Assimes TL, Thompson JR, et al. Large-scale association analysis identifies new risk loci for coronary artery disease. Nat Genet. 2013;45:25-33.
69. Webb TR, Erdmann J, Stirrups KE, Stitziel NO, Masca NG, Jansen H, et al. Systematic evaluation of pleiotropy identifies 6 further loci associated with coronary artery disease. J Am Coll Cardiol. 2017;69:823-36.
70. Wannamethee SG, Shaper AG, Lennon L, Morris RW. Metabolic syndrome vs Framingham Risk Score for prediction of coronary heart disease, stroke, and type 2 diabetes mellitus. Arch Intern Med. 2005;165:2644-50.
71. Miller RJH, Gransar H, Rozanski A, Dey D, Al-Mallah M, Chow BJW, et al. Simplified approach to predicting obstructive coronary disease with integration of coronary calcium: Development and external validation. J Am Heart Assoc. 2023;12:e031601.
72. Cao RY, Yang J, Zheng Y, Li H, Zhao Q, Ding Y, et al. The potential value of Copeptin and Pentraxin3 for evaluating the severity of coronary stenosis in patients with coronary artery disease. Clin Biochem. 2021;87:32-8.
73. Xu L, Fu T, Wang Y, Ji N. Diagnostic value of peripheral blood miR-296 combined with vascular endothelial growth factor B on the degree of coronary artery stenosis in patients with coronary heart disease. J Clin Ultrasound. 2023;51:520-9.
74. Jiang Y, Du T. Relation of circulating lncRNA GAS5 and miR-21 with biochemical indexes, stenosis severity, and inflammatory cytokines in coronary heart disease patients. J Clin Lab Anal. 2022;36:e24202.
75. Cho AR, Lee SY. Biomarkers and their relative contributions to identifying coronary artery stenosis based on coronary computed tomography angiography in asymptomatic adults. Clin Chim Acta. 2019;499:128-33.
76. McCarthy CP, Neumann JT, Michelhaugh SA, Ibrahim NE, Gaggin HK, Sörensen NA, et al. Derivation and external validation of a high-sensitivity cardiac troponin-based proteomic model to predict the presence of obstructive coronary artery disease. J Am Heart Assoc. 2020;9:e017221.
77. Writing Committee Members; Gulati M, Levy PD, Mukherjee D, Amsterdam E, Bhatt DL, Birtcher KK, et al. 2021 AHA/ACC/ASE/CHEST/SAEM/SCCT/SCMR Guideline for the Evaluation and Diagnosis of Chest Pain: A Report of the American College of Cardiology/American Heart Association Joint Committee on Clinical Practice Guidelines. J Am Coll Cardiol. 2021;78:e187-e285.
78. Fihn SD, Gardin JM, Abrams J, Berra K, Blankenship JC, Dallas AP, et al. 2012 ACCF/AHA/ACP/AATS/PCNA/SCAI/STS Guideline for the diagnosis and management of

patients with stable ischemic heart disease: a report of the American College of Cardiology Foundation/American Heart Association Task Force on Practice Guidelines, and the American College of Physicians, American Association for Thoracic Surgery, Preventive Cardiovascular Nurses Association, Society for Cardiovascular Angiography and Interventions, and Society of Thoracic Surgeons. J Am Coll Cardiol. 2012;60:e44-e164.

79. Tiwari N, Margapuri J, Katamreddy A, Jubbal S, Madan N. Diagnostic accuracy of cardiac testing for coronary artery disease in potential liver transplant recipients: A systematic review and meta-analysis. Int J Cardiol Heart Vasc. 2021;32:100714.
80. Greenwood JP, Maredia N, Younger JF, Brown JM, Nixon J, Everett CC, et al. Cardiovascular magnetic resonance and single-photon emission computed tomography for diagnosis of coronary heart disease (CE-MARC): a prospective trial. Lancet. 2012;379:453-60.
81. Chen X, Zhao J, Cai Q, Chen R, Wu W, Wang P, et al. Relationship between Coronary Artery Calcium Score and Coronary Stenosis. Cardiol Res Pract. 2023;2023:5538111.
82. Nurmohamed NS, Bom MJ, Jukema RA, de Groot RJ, Driessen RS, van Diemen PA, et al. AI-guided quantitative plaque staging predicts long-term cardiovascular outcomes in patients at risk for atherosclerotic CVD. JACC Cardiovasc Imaging. 2024;17:269-80.
83. Lawton JS, Tamis-Holland JE, Bangalore S, Bates ER, Beckie TM, Bischoff JM, et al. 2021 ACC/AHA/SCAI Guideline for Coronary Artery Revascularization: A Report of the American College of Cardiology/American Heart Association Joint Committee on Clinical Practice Guidelines. Circulation. 2022;145:e18-e114.
